# Small molecule agonist TPC2-A1-N increases intracellular Ca^2+^ independent of two-pore channels

**DOI:** 10.1016/j.jbc.2025.110576

**Published:** 2025-08-08

**Authors:** Robert T. Mallmann, Marlene C. Gonzalez Mantuano, Katharina Polomski, Julian Knerr, Norbert Klugbauer

**Affiliations:** Institute for Experimental and Clinical Pharmacology and Toxicology, Medical Faculty, Albert-Ludwigs-University Freiburg, Freiburg, Germany

**Keywords:** calcium intracellular release, endosome, endoplasmic reticulum, ion channel, intracellular trafficking, two-pore channel, TPC, TPC-A1-N, NAADP, Ca^2+^ measurement, endo-lysosomal system

## Abstract

Two-pore channels (TPCs) constitute a small family of cation channels expressed in endo-lysosomal compartments. TPCs have been characterized as important constituents controlling Ca^2+^-mediated vesicular membrane fusion and fission, thereby regulating intracellular organelle trafficking. Two activators, nicotinic acid adenine dinucleotide phosphate and phosphatidylinositol-3,5-bisphosphate, induce ion flux through TPCs. The membrane-permeable small molecule activators TPC2-A1-N and TPC2-A1-P have been identified and postulated to mimic their action and to discriminate for a preferential selectivity either for Ca^2+^ or for Na^+^. This was observed only for TPC2 and was independent of nicotinic acid adenine dinucleotide phosphate–binding proteins. Here, we applied TPC2-A1-N and measured intracellular increase of Ca^2+^ and Na^+^ in mouse embryonic fibroblast, HeLa, and J774 cells. TPC2-A1-N did not only increase Ca^2+^ levels in WT but also in all cells with genetically inactivated TPCs. Depletion of Ca^2+^ from the endoplasmic reticulum (ER) *via* thapsigargin caused a massive reduction of the TPC2-A1-N induced Ca^2+^ elevation in all cell lines, indicating that ER plays a key role in this context. Furthermore, our results point to an inositol triphosphate receptor–independent TPC2-A1-N mediated Ca^2+^ release. Ca^2+^ depletion from ER was also observed by using an ER-targeted GCaMP6 construct. TPC2-A1-N also raised Na^+^ levels in mouse embryonic fibroblast cells deficient for TPC1 and TPC2. In summary, our results suggest that TPC2-A1-N induced Ca^2+^ and Na^+^ signals are independent of any TPC and that ER represents the major source of Ca^2+^.

Two-pore channel (TPC) proteins constitute a small family of endo-lysosomal cation channels (1, 2, 3). Due to their sequence homology and overall transmembrane and domain structure, they are considered to belong to the superfamily of voltage-gated ion channels (4, 5). TPCs probably represent evolutionary intermediates between single and four domain comprising channels. In humans and rodents, there are two isoforms, TPC1 and TPC2, demonstrating a preferential, but also partially overlapping expression pattern within endo-lysosomal compartments (6). Functionally, they have been described as nonselective cation channels with a certain focus on Ca^2+^ and Na^+^ permeability (7, 8, 9, 10, 11, 12, 13). For instance, TPCs provide a locally and temporally restricted Ca^2+^ efflux from endo-lysosomal vesicles, thereby controlling membrane fusion and fission processes. Accordingly, TPCs are thought to be involved in the regulation of endocytosis, recycling, and degradation, as well as in vesicle trafficking, sorting, and fusion (8, 14, 15).

The activation of TPCs depends on membrane depolarization but also on the binding of the lipid phosphatidylinositol-3,5-bisphosphate (PI(3,5)P_2_) or on the presence of the intracellular messenger nicotinic acid adenine dinucleotide phosphate (NAADP) (7, 10, 11, 16). There is a huge body of literature describing the different ways to investigate TPCs; however, due to their localization in membranes of the endo-lysosomal system those techniques work indirectly (9, 10, 11, 16, 17, 18, 19, 20, 21). These methods analyze TPCs either in lipid bilayers, in enlarged vacuoles formed by endo-lysosomal membrane compartments, or take advantage of mutated TPCs rerouting them to the plasma membrane. The latter techniques allow for the application of patch clamp either by investigating TPCs in artificially fused and enlarged endo-lysosomal membrane compartments or in the non-native lipid environment of the plasma membrane. Due to the lack of more suitable approaches, most functional analyses have been performed in this way. In fact, it is clear that all techniques represent a compromise that relies on a series of assumptions. For sure the pH values differ between early, late, and recycling endosomes or lysosomes and the artificial organelles used for patch clamp. Additionally, and of critical significance, the protein or subunit composition of TPC complexes might be altered in those artificial compartments. Especially in the context of the second messenger NAADP, the presence of NAADP-binding proteins is questioned and one might expect altered activation mechanisms or a lack of crucial regulatory proteins (12, 13, 22, 23, 24). Furthermore, the spectrum of phosphatidylinositol-mono- and di-phosphate derivatives within the plasma membrane and membranes of the endo-lysosomal compartments may have drastic effects on TPC activation (25). The latter are dominated by 3′-phosphoinositides with phosphatidylinositol-3-phosphate as signature lipid of early endosomes and PI(3,5)P_2_ as prevalent lipid of late endosomes (26). This is of particular importance since PI(3,5)P_2_ is one of the most frequently used activators of TPCs, although it is present only in distinct regions of endo-lysosomal membranes.

The methods used for characterizing TPCs also do not take into account the existence of contact sites between endo-lysosomal and endoplasmic reticulum (ER) membranes. Ca^2+^ homeostasis in general or Ca^2+^ release from these stores might strongly rely on the bidirectional Ca^2+^ exchange between those compartments (27). In fact, this was already demonstrated for Ca^2+^-dependent histamine release from mast cells, where TPC1 is important for endo-lysosomal Ca^2+^ uptake and filling of ER Ca^2+^ stores *via* a bidirectional Ca^2+^ exchange (28).

Two membrane-permeable small molecule activators, TPC2-A1-N and TPC2-A1-P, have been identified and described to mimic the action of commonly used TPC activators NAADP and PI(3,5)P_2_ exclusively for TPC2. The identification of the two substances strongly contributed to the ongoing discussion about Ca^2+^ and Na^+^ selectivity of TPCs. By using above mentioned techniques it was suggested that application of the NAADP mimetic TPC2-A1-N results in a higher relative Ca^2+^ permeability, whereas the PI(3,5)P_2_ mimetic TPC2-A1-P favors Na^+^ over Ca^2+^ permeability (21).

The aim of our study was to overcome and to take into account the limitations of frequently used TPC measurements by using a protocol with the primary objective to characterize TPCs in their native endo-lysosomal membrane compartments. Therefore, we did not mutate TPCs, did not reroute them to the plasma membrane and did not create artificially enlarged organelles, instead, we kept TPCs in their native membrane setting and activated them by addition of the NAADP mimetic TPC2-A1-N. The increase of intracellular Ca^2+^ and Na^+^ was measured by Fura-2 and CoroNa Green, respectively. Remarkably, all cell lines tested with a genetic deletion of either TPC1 or TPC2 and even both TPCs demonstrated a robust increase of intracellular Ca^2+^. This result strongly indicates that TPC2-A1-N increases intracellular Ca^2+^ independent of any TPC.

## Results

### TPC2-A1-N induced Ca^2+^ and Na^+^ increase in mouse embryonic fibroblast (MEF) WT and TPC-deficient cells

**Figure 1:**
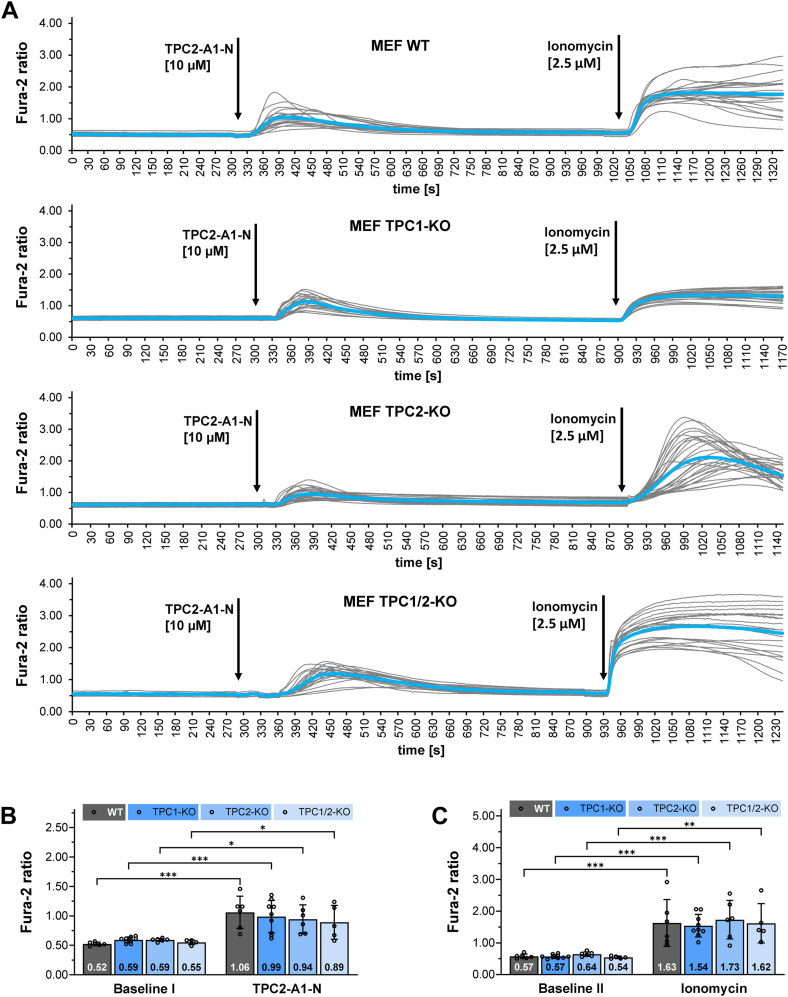
**TPC2-A1-N induced Ca^2+^ increase in MEF WT and TPC-deficient cells.***A*, single track Ca^2+^ recordings from 23 Fura-2 loaded WT, 24 TPC1-, 28 TPC2- and 20 TPC1/2-double KO MEF cells (*gray lines* correspond to each individual cell; mean Fura-2 ratio is shown as *blue line*). Fura-2 ratios were gathered in Ca^2+^-free Hank's balanced salt solution buffer. Following incubation with TPC2-A1-N 2.5 μM ionomycin was added. *B*, comparison of Fura-2 ratios at baseline levels and maximal Fura-2 ratios following treatment with TPC2-A1-N. *C*, comparison of Fura-2 ratios at baseline levels and maximal Fura-2 ratios after application of ionomycin. *A*-*C*,)mean values represent the average maximal Fura-2 ratios of N experiments per cell line. *B* and *C*, (MEF-WT (N = 6) MEF-TPC1-KO (N = 9) MEF-TPC2-KO (N = 6) MEF-TPC1/2-KO (N = 5); data are presented as mean ± SD. Two-way repeated measures ANOVA followed by Bonferroni’s multiple comparisons test. N = number of experiments; ∗*p* < 0.05, ∗∗*p* < 0.01, and ∗∗∗*p* < 0.001. TPC, two-pore channel.

We aimed to study TPCs in their native endo-lysosomal compartments, therefore, we measured the increase of Ca^2+^ following application of membrane permeable small ligand TPC2-A1-N, a substance described as NAADP mimetic for TPC2 (21). In a first series of experiments, we used an entire set of mouse embryonic fibroblasts including mouse embryonic fibroblast (MEF) WT, TPC1- and TPC2-single-KO and TPC1/2 double-KO cells. MEF cells were derived from transgenic mouse lines deficient either for TPC1 or TPC2, generation, and verification of knock out is described in references (8, 29). TPC1/2 double-KO MEF cell line was generated by an additional CRISPR-Cas9–mediated TPC1 inactivation in TPC2 KO cells (14). Successful deletion was verified by sequence analysis of genomic DNA and by Western blot and is shown in supplemental items of reference (14). Cells were stimulated by addition of 10 μM or 25 μM TPC2-A1-N in the absence of extracellular Ca^2+^. Increase of intracellular Ca^2+^ was measured by Fura-2. As expected, TPC2-A1-N caused an increase of Ca^2+^ in WT MEF cells (Fig. 1*A* and supporting information Fig. S1*A*). The size of the observed Ca^2+^ signal was fairly large; therefore, the contribution of different Ca^2+^ stores was investigated further on in our study. To exclude potential bias of dimethylsulfoxide (DMSO), control experiments were performed using the same DMSO concentration as for 25 μM TPC2-A1-N studies (Fig. S3, *A* and *B*).

However, to our surprise a statistically significant rise in Ca^2+^ was observed in all TPC-deficient MEF cells (Figs. 1, *A*, *B* and S1*B*). In the further course of the experiment, cells were treated with ionomycin to release Ca^2+^ from all intracellular Ca^2+^ stores that were not affected by TPC2-A1-N. Data presented in Figure 1*C* and corresponding Fig. S1*C* show a significant increase of Ca^2+^ above baseline levels. This result indicated integrity of plasma and intracellular membranes after addition of TPC2-A1-N. Additionally, we calculated the slopes of Fura-2 ratios over time at the half-maximum values following application of TPC2-A1-N. None of the TPC-deficient MEF cells differed from WT cells with respect to this kinetic parameter (Fig. S2*A*).

**Figure 2:**
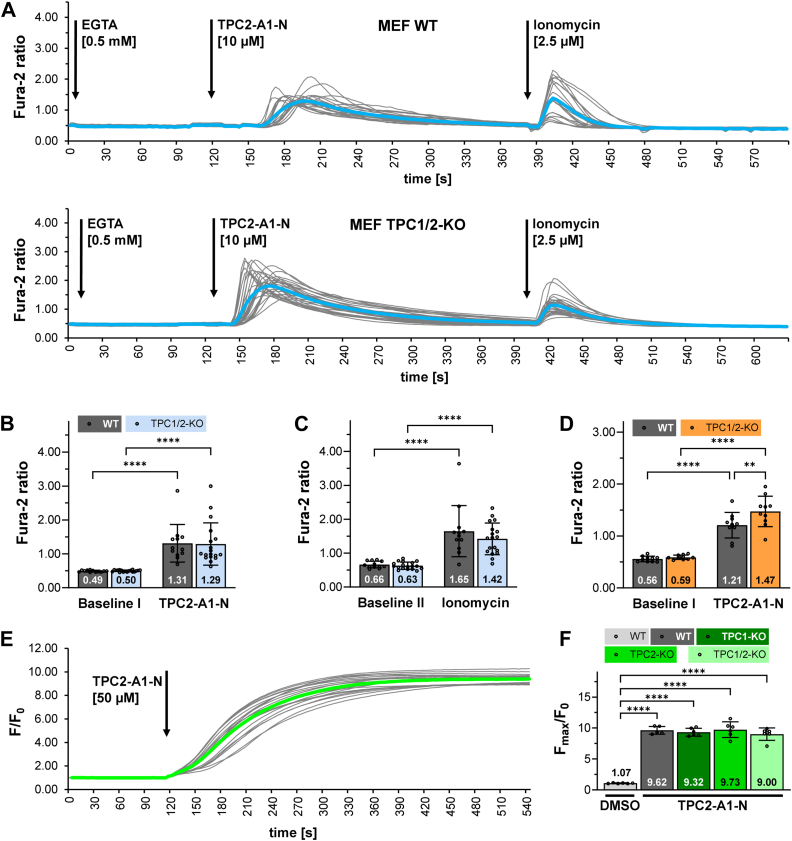
**TPC2-A1-N induced Ca^2+^ and Na^+^ increase in MEF WT and TPC-deficient cells.***A*, single track recordings from 20 Fura-2 loaded WT and 19 TPC1/2-double-KO MEF cells (*gray lines* correspond to each individual cell; mean Fura-2 ratio is shown as *blue line*). Cells were incubated first in Hank's balanced salt solution (HBSS) buffer supplemented with 2 mM CaCl_2_. Fura-2 ratios were analyzed after buffer exchange to Ca^2+^ free HBSS and addition of EGTA. Following incubation with TPC2-A1-N, 2.5 μM ionomycin was added. *B*, comparison of Fura-2 ratios at baseline levels and maximal Fura-2 ratios following treatment with TPC2-A1-N. *C*, comparison of Fura-2 ratios at baseline levels and maximal Fura-2 ratios after application of ionomycin. *D*, maximal Ca^2+^ release of MEF WT and TPC1/2-KO after treatment with TPC2-A1-N in HBSS buffer containing 2 mM CaCl_2_. *E*, single track Na^+^ recordings from 18 CoroNa-Green loaded TPC1/2-KO MEF cells (*gray lines* correspond to each individual cell; mean CoroNa-Green fluorescence intensity is shown as *green line*). CoroNa-Green fluorescence was analyzed in HBSS supplemented with 2 mM Ca^2+^. *F*, maximal changes of CoroNa-Green fluorescence after incubation with TPC2-A1-N. *B*-*D*, mean values represent the average maximal Fura-2 ratios of N experiments per cell line. *B* and *C*, Ca^2+^-free and EGTA protocol: MEF-WT (N = 12) MEF-TPC1/2KO (N = 18). *D*, 2 mM CaCl_2_ extracellular protocol: MEF-WT (N = 10) MEF-TPC1/2KO (N = 10); data are presented as mean ± SD. Two-way repeated measures ANOVA followed by Bonferroni’s multiple comparisons test. *F*, mean values represent the average maximal CoroNa-Green fluorescence of N experiments per cell line and treatment. MEF WT (dimethylsulfoxide-control N = 6) MEF-WT (N = 5) MEF-TPC1-KO (N = 6) MEF-TPC2 (N = 6) MEF-TPC1/2KO (N = 6); data are presented as mean ± SD. One-way ANOVA and Tukey’s multiple comparisons test; ∗∗*p* < 0.01, ∗∗∗*p* < 0.001, and ∗∗∗∗*p* < 0.0001. TPC, two-pore channel.

The next set of experiments was designed to exclude any potential effect of residual extracellular Ca^2+^ traces. A total of 0.5 mM EGTA was added in the bath solution and Ca^2+^ measurement was repeated with 10 μM TPC2-A1-N using WT and TPC1/2 double KO MEF cells. Again, TPC2-A1-N increased intracellular Ca^2+^ levels in both cell lines (Fig. 2, *A* and *B*). Control experiments with ionomycin confirmed integrity of cellular membranes and presence of residual Ca^2+^ in TPC2-A1-N nontargeted organelles (Fig. 2, *A* and *C*). The same kinetic analysis was performed for this set of experiments as before, but again, there was no difference in the slopes of the Fura-2 ratios over time between WT and TPC1/2-KO cells (Fig. S2*B*). We further studied a potential reinforcing effect of extracellular Ca^2+^ by addition of 2 mM CaCl_2_ to the bath solution. In WT and TPC1/2 double KO cells, incubation with 10 μM TPC2-A1-N significantly increased intracellular Ca^2+^ in both lines and remarkably, the rise of Ca^2+^ was higher in TPC1/2 double KO cells (Fig. 2*D*).

The small molecule activator TPC2-A1-N has been postulated to represent an NAADP mimetic that favors the release of Ca^2+^ over Na^+^ (21). In order to test for a potential intracellular increase of Na^+^ in our set of MEF cells, we took advantage of the Na^+^-ion indicator CoroNa-Green. Addition of 50 μM TPC2-A1-N significantly increased CoroNa-Green fluorescence in WT and all KO MEF cell lines indicating a rise of cytosolic Na^+^ (Fig. 2, *E* and *F*).

### TPC2-A1-N induced Ca^2+^ release from intracellular stores in WT and TPC1/2 double KO J774 and HeLa cells

**Figure 3:**
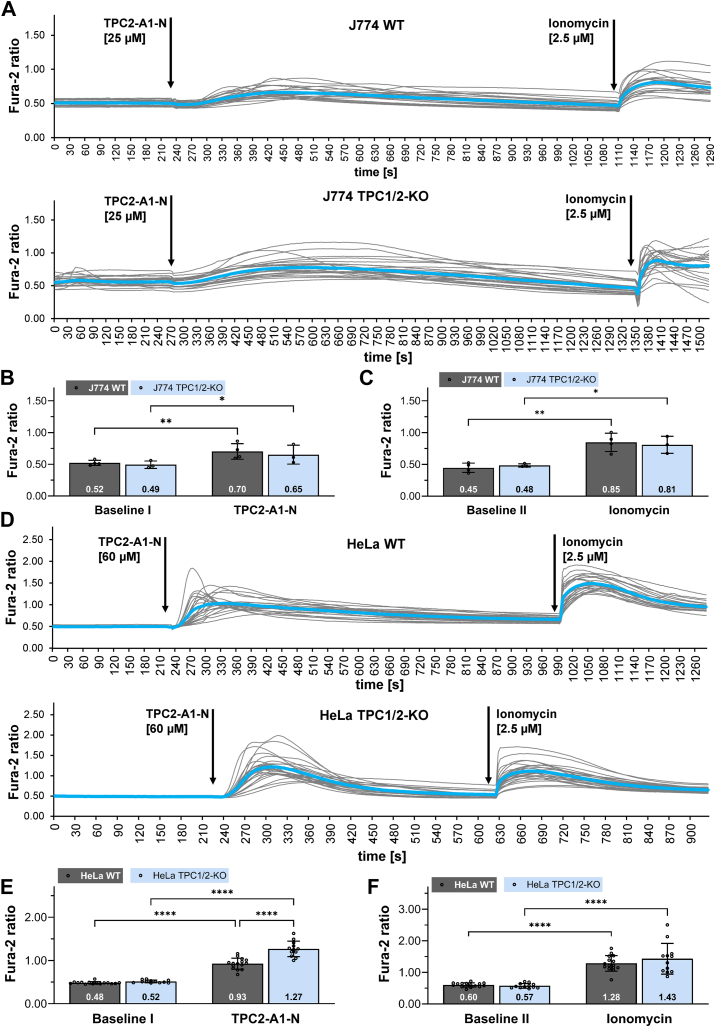
**TPC2-A1-N induced Ca^2+^ release from intracellular stores in WT and TPC1/2 double KO J774 and HeLa cells.***A*, single track recordings from 21 Fura-2 loaded WT and 24 TPC1/2-KO J774 cells (*gray lines* correspond to each individual cell; mean Fura-2 ratio is shown as *blue line*). Fura-2 ratios were gathered in Ca^2+^-free Hank's balanced salt solution buffer. Following incubation with TPC2-A1-N, ionomycin was added. *B*, comparison of Fura-2 ratios at baseline levels and maximal Fura-2 ratios following treatment with TPC2-A1-N. *C*, comparison of Fura-2 ratios at baseline levels and maximal Fura-2 ratios after application of ionomycin. *D*, single track recordings from 22 Fura-2 loaded WT and 24 TPC1/2-KO HeLa cells (*gray lines* correspond to each individual cell; mean Fura-2 ratio is shown as *blue line*). Fura-2 ratios were gathered in Ca^2+^-free Hank's balanced salt solution buffer. Following incubation with TPC2-A1-N, 2.5 μM ionomycin was added. *E*, comparison of Fura-2 ratios at baseline levels and maximal Fura-2 ratios following treatment with TPC2-A1-N. *F*, comparison of Fura-2 ratios at baseline levels and maximal Fura-2 ratios after application of ionomycin. Mean of the average maximal Fura-2 ratios of N experiments per cell line; J774-WT (N = 4) J774-TPC1/2KO (N = 3); HeLa-WT (N = 15); HeLa-TPC1/2-KO (N = 12); data are presented as mean ± SD. Two-way repeated measures ANOVA followed by uncorrected Fisher’s least significant difference (*B* and *C*) or Bonferroni’s multiple comparisons (*E* and *F*) test; ∗*p* < 0.05, ∗∗*p* < 0.01, and ∗∗∗∗*p* < 0.0001. TPC, two-pore channel.

TPCs have been shown to represent crucial regulators for endomembrane modelling processes, particularly during reformation of lysosomes from the phagosome membrane network. Here, TPCs control release of membrane tension that permits deformation of the limiting phagolysosome membrane (30). Therefore, we investigated Ca^2+^ release in the macrophage cell line J774 by applying the CRISPR-Cas9 system to inactivate both TPCs. SI Fig. S4, *A*–*C* demonstrate successful genetic inactivation by sequence analysis of genomic DNA and Western blot. Sequence analysis confirmed a 1 bp deletion in exon 5 of both TPC1 alleles and a 1 bp addition in exon 4 of both TPC2 alleles. Application of the Ca^2+^ measurement protocol in the absence of extracellular Ca^2+^ is shown in Figure 3, *A*–*C*. Again, we observed a rise of intracellular Ca^2+^ after application of 25 μM TPC2-A1-N in WT but also in TPC1/2 double-KO J774 cells. This result confirms our basic finding obtained with MEF cells, namely an increase of intracellular Ca^2+^ independent of any TPC.

Finally, we deleted TPCs in a third cell line, human HeLa cells, to demonstrate that our observations are not restricted to some murine cell lines. CRISPR-Cas9–mediated targeting of TPC1 and TPC2 genes resulted in a complete knockout of both TPCs. Sequence analysis confirmed the same mutations in both alleles of TPC1 exon 8 and both alleles of TPC2 exon 4 (Fig. S5, *A* and *B*). Western blot analysis indicated that expression levels of TPC1 and TPC2 were widely differing in HeLa cells with a clear band visible for TPC1, but not for TPC2. To confirm detection of human TPC2 protein by the antibody, we transfected HeLa double KO cells with a plasmid encoding human TPC2. Western blot analysis of transfected cells resulted in a protein band of correct size (Fig. S5*C*).

We applied 60 μM TPC2-A1-N in our Ca^2+^ measurement protocol to stimulate HeLa cells in the absence of extracellular Ca^2+^. Remarkably, TPC2-A1-N even resulted in significantly higher Ca^2+^ levels in TPC1/2 double KO than in WT cells (Fig. 3, *D* and *E* and ionomycin control in Fig. 3*F*). To exclude potential stimulating effects of DMSO, a control experiment was performed using the same DMSO concentration as for the 60 μM TPC2-A1-N application (Fig. S3, *C* and *D*).

### Residual TPC2-A1-N and thapsigargin evoked Ca^2+^ signals in MEF cells following application of either thapsigargin or TPC2-A1-N

TPCs have also been shown to be essential for the crosstalk and balance of Ca^2+^ between compartments of the endo-lysosomal system and the ER (31). In immune cells, TPCs contribute to Ca^2+^-dependent release processes for a large number of secretory granules. For instance, TPC1 deficiency leads to an augmented histamine release in murine mast cells and *in vivo* to an accelerated histamine-induced vasodilation (28). Thus, TPC1 has a crucial role not only to increase cytoplasmic Ca^2+^ but probably also for the uptake of endo-lysosomal Ca^2+^ and for filling of ER Ca^2+^ stores. Given these facts, we investigated the effects of TPC2-A1-N in TPC-deficient cell lines after depleting ER Ca^2+^ stores by thapsigargin. Our protocol starts with the addition of 2.5 μM thapsigargin, followed by either 25 μM (MEF) or 60 μM (HeLa) TPC2-A1-N and finally 2.5 μM ionomycin.

**Figure 4:**
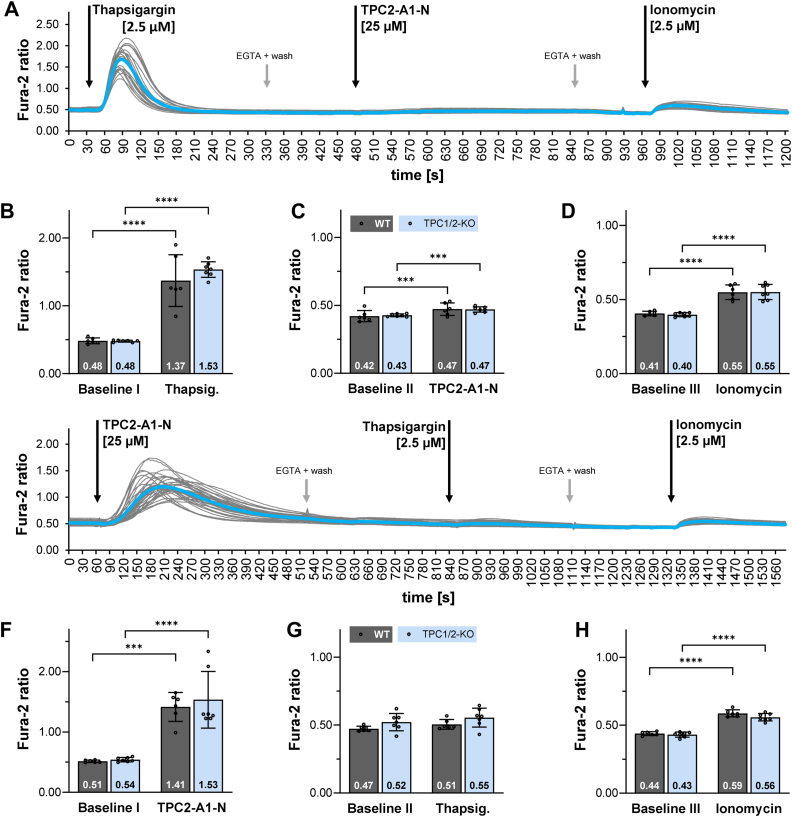
**Residual TPC2-A1-N and thapsigargin evoked Ca^2+^ signals in MEF cells following application of thapsigargin or TPC2-A1-N.***A*, single track recordings from 22 Fura-2 loaded MEF-TPC1/2-KO cells (*gray lines* correspond to each individual cell; mean Fura-2 ratio is shown as *blue line*). Cells were incubated first in Hank's balanced salt solution (HBSS) supplemented with 2 mM CaCl_2_. Fura-2 ratios were analyzed following buffer exchange to Ca^2+^-free HBSS. EGTA (0.5 mM) and a washing step with Ca^2+^-free HBSS buffer were applied between each stimulation step. Following incubation with thapsigargin and TPC2-A1-N, 2.5 μM ionomycin was added. *B*, comparison of Fura-2 ratios at baseline levels and maximal Fura-2 ratios following treatment with 2.5 μM thapsigargin. *C*, comparison of Fura-2 ratios at baseline levels and maximal Fura-2 ratios following treatment with TPC2-A1-N. *D*, comparison of Fura-2 ratios at baseline levels and maximal Fura-2 ratios after application of ionomycin. *E*, single track recordings from 38 Fura-2 loaded MEF-TPC1/2-KO cells (*gray lines* correspond to each individual cell; mean Fura-2 ratio is shown as *blue line*). Cells were incubated first in HBSS supplemented with 2 mM CaCl_2_. Fura-2 ratios were analyzed following buffer exchange to Ca^2+^-free HBSS. EGTA (0.5 mM) and a washing step with Ca^2+^-free HBSS buffer were applied between each stimulation step. Following incubation with TPC2-A1-N and thapsigargin, 2.5 μM ionomycin was added. *F*, comparison of Fura-2 ratios at baseline levels and maximal Fura-2 ratios following treatment with 25 μM TPC2-A1-N. *G*, comparison of Fura-2 ratios at baseline levels and maximal Fura-2 ratios following treatment with 2.5 μM thapsigargin. *H*, comparison of Fura-2 ratios at baseline levels and maximal Fura-2 ratios after application of ionomycin. *B*-*D*, MEF-WT (N = 6) MEF-TPC1/2KO (N = 7). *F*-*H*, MEF-WT (N = 6) MEF-TPC1/2KO (N = 7); N = number of experiments. Data are presented as mean ± SD. Two-way repeated measures ANOVA followed by Bonferroni’s multiple comparisons test; ∗∗∗*p* < 0.001, ∗∗∗∗*p* < 0.0001. buffer exchange.

Figure 4 shows the results of this protocol for WT and TPC1/2 double KO MEF and Fig. S6 for HeLa cells, respectively. As expected, incubation with thapsigargin released Ca^2+^ from the ER and significantly increased cytosolic Ca^2+^ levels in all cell lines (Figs. 4, *A*, *B* and S6, *A*, *B*). Upon return to baseline Ca^2+^ levels, the addition of TPC2-A1-N resulted only in a marginally, but still significant rise of Ca^2+^ in all cell lines tested (Figs. 4, *A*, *C* and S6, *A*, *C*). Finally, application of ionomycin resulted in a comparable release of Ca^2+^ in WT and TPC1/2 double KO MEF and HeLa cells, indicating for Ca^2+^ stores not targeted by TPC2-A1-N or thapsigargin (Figs. 4, *A*, *D* and S6, *A*, *D*).

In the next set of experiments, we changed the order of the substances to check for a comparable effect if TPC2-A1-N is applied first, followed by thapsigargin and finally ionomycin. Figure 4*E* shows the results for TPC1/2 double KO MEF cells. TPC2-A1-N strongly increased Ca^2+^ levels in WT and TPC1/2 double KO cells (Fig. 4*F*), but the following addition of thapsigargin resulted in a rather marginal rise of Ca^2+^ levels (Fig. 4*G*). This *vice versa* experiment also suggests that the primary Ca^2+^ store targeted by TPC2-A1-N is the ER. Finally, application of ionomycin resulted in a comparable release of Ca^2+^ in WT and TPC1/2 double KO cells, indicating for Ca^2+^ stores not targeted by TPC2-A1-N or thapsigargin (Fig. 4*H*).

In order to uncover a potential role of the inositol triphosphate (IP_3_) receptor for the action of TPC2-A1-N, we established a protocol based on the application of the Ca^2+^ releasing substance histamine and 2-2-aminoethoxydiphenyl borate (APB) as an inhibitor of IP_3_ receptors. Addition of 10 μM histamine increased intracellular Ca^2+^ levels in WT and TPC1/2 double KO HeLa cells *via* activation of histamine-1 and subsequently IP_3_ receptors (Fig. S7, *A*–*D*). Application of histamine typically causes an initial and uniform rise of Ca^2+^, followed by rather irregular oscillations (32).

Pretreatment with 100 μM 2-APB blocked this histamine-induced Ca^2+^ increase (Fig. S8, *A* and *B*). The following application of TPC2-A1-N still showed a moderate, but statistically significant increase of Ca^2+^ (Fig. S8, *A* and *C*). This result indicates that TPC2-A1-N causes a rise of intracellular Ca^2+^ at least in part independent of IP_3_ receptors. Ionomycin was used as a control (Fig. S8*D*). When we reversed the order of activators following application of 100 μM 2-APB and started with TPC2-A1-N, a significant Ca^2+^ rise occurred (Fig. S8, *E* and *F*). The following addition of histamine did no further increase intracellular Ca^2+^ (Fig. S8, *E* and *G*). However, the addition of ionomycin increased intracellular Ca^2+^ levels, indicating the presence and integrity of residual Ca^2+^ stores (Fig. S8, *E* and *H*). In summary, these experiments demonstrate that TPC2-A1-N acts at least in part independent of IP_3_ receptors.

### Decrease of luminal ER Ca^2+^-concentration in MEF cells after addition of thapsigargin or TPC2-A1-N

**Figure 5:**
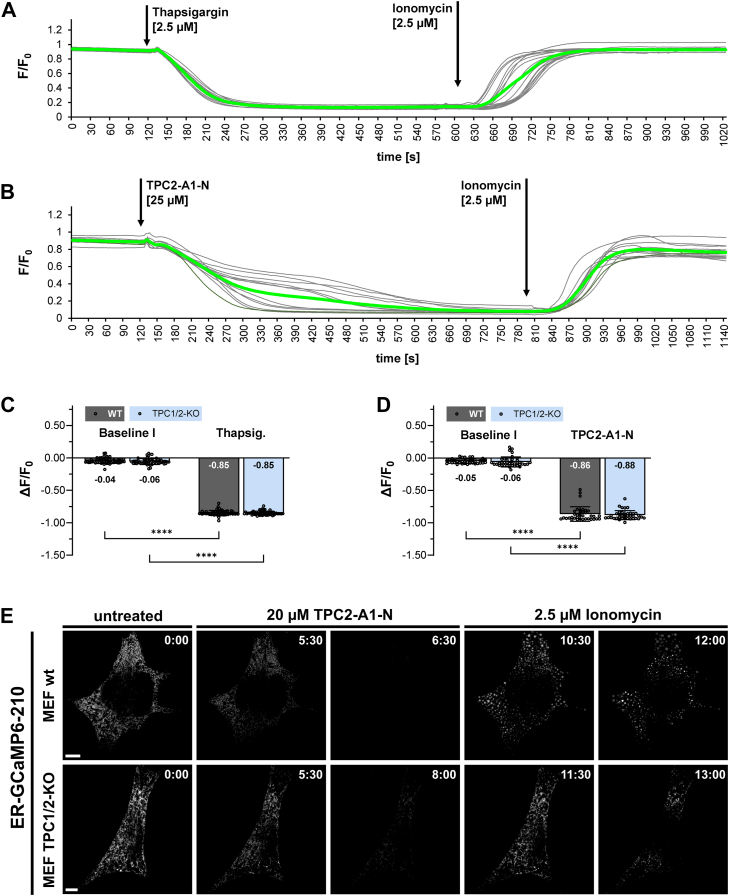
**Decrease of luminal ER Ca^2+^-concentration in MEF cells after addition of thapsigargin or TPC2-A1-N.***A* and *B*, single track recordings of (*A*) 16 and (*B*) 12 TPC1/2-KO MEF cells transfected with ER-GCaMP6-210 (*gray lines* correspond to each individual cell; mean ER-GCaMP6-210 fluorescence intensity is shown as *green line*). Cells were treated with (*A*) 2.5 μM thapsigargin or (*B*) 25 μM TPC2-A1-N. ER-GCaMP6-210 fluorescence was analyzed in Hank's balanced salt solution supplemented with 2 mM Ca^2+^. *C* and *D*, changes in fluorescence at baseline and minimal ΔF/F0 values following treatment with (*C*) 2.5 μM thapsigargin (MEF WT N = 40 cells, MEF TPC1/2-KO N = 41 cells) or (*D*) 25 μM TPC2-A1-N (MEF WT N = 36 cells, MEF TPC1/2-KO N = 38 cells). Data are presented as mean ± SD. Two-way repeated measures ANOVA followed by Bonferroni’s multiple comparisons test (*C* and *D*); ∗∗∗∗*p* < 0.0001. *E*, example images from airyscan time-lapse videos of WT and TPC1/2-KO MEF cells transiently transfected with ER-GCaMP-210 (*white*) and treated with TPC2-A1-N and ionomycin. Image series shows initially untreated cells (0–2 min), addition of 20 μM TPC2-A1-N until ER-GCaMP-210-fluorescence was almost undetectable (5:00–9:30 min) and addition of 2.5 μM ionomycin (10:00 min-end). The scale bar represents 5 μm; time: mm:ss. ER, endoplasmic reticulum; TPC, two-pore channel.

Since our results point to an essential role of the ER for TPC2-A1-N–induced Ca^2+^ release, we intended to directly track the decrease of Ca^2+^ from the ER. The ER-GCaMP6-210 construct is an ideal tool to follow the dynamics of luminal Ca^2+^ concentrations by live-cell imaging. The affinity of the ER-GCaMP6-210 construct for Ca^2+^ matches that for resting ER Ca^2+^ levels (33). First, we applied thapsigargin to WT and TPC1/2-KO MEF cells to check for the time course of ER-GCaMP6-210 fluorescence decrease as a response to Ca^2+^ efflux from the ER (Fig. 5, *A* and *C*). The following addition of ionomycin in Ca^2+^ containing Hank's balanced salt solution (HBSS) buffer caused an increase of the ER-GCaMP6-210 fluorescence intensity (Fig. 5*A*). The same protocol was applied to WT and TPC1/2-KO MEF cells but with TPC2-A1-N and resulted in a comparable, but slower decrease of ER-GCaMP6-210 fluorescence (Fig. 5*B*). Figure 5, *C* and *D* show statistical analysis of these experiments and indicate that there are no differences between WT and TPC1/2-KO cells with respect to the two protocols and demonstrate similar ER-GCaMP6-210 fluorescence decreases for thapsigargin and TPC2-A1-N. The analysis of slopes at the half-minimal ΔF/Δt values following addition of either TPC2-A1-N or thapsigargin revealed no differences between MEF WT and TPC1/2-KO cells, but a significant slower decrease for TPC2-A1-N than for thapsigargin (Fig. S9). Figure 5*E* and Fig. S10 and supporting videos V1 to V4visualize time course of ER-GCaMP6-210 fluorescence in time-lapse videos.

In our final set of experiments, we transfected MEF WT and TPC1/2-KO cells with a plasmid encoding TPC1-GCaMP7f, that is, GCaMP fused to the C terminus of TPC1. This construct localizes the Ca^2+^ sensor part to the cytosolic side of endo-lysosomal compartments. The addition of 20 μM TPC2-A1-N resulted in a fast increase of the GCaMP7f fluorescence and did not reveal any differences in the ΔF/F0 values between the genotypes (Fig. S11 and supporting videos V5 and V6).

In summary, all our Ca^2+^ and Na^+^ measurements in an entire set of WTs and TPC1 and TPC2 single and TPC1/2 double KO cells indicate that the small molecule activator TPC2-A1-N increases the intracellular concentrations of both ions independent of TPCs.

## Discussion

In our study, we investigated the effect of the small molecule activator TPC2-A1-N on the increase of intracellular Ca^2+^ and Na^+^ in murine and human cell lines. TPC2-A1-N has been described as an activator of the TPC2 and as a mimetic of NAADP causing the release of Ca^2+^ from endo-lysosomal stores (21). Furthermore, TPC2-A1-N and a second small molecule activator of TPC2, TPC2-A1-P have been postulated to bias ion channel selectivity, namely a preferential permeability of Ca^2+^ over Na^+^ for TPC2-A1-N and *vice versa* for TPC2-A1-P.

As expected, TPC2-A1-N significantly and rapidly increased intracellular Ca^2+^ levels in all WT cells investigated. However, to our surprise the same rise in Ca^2+^ was also observed in the same cell lines lacking TPC2. Since we intended to study both members of the TPC family, we also inactivated TPC1 and established TPC1/2 double KO cell lines. With respect to intracellular Ca^2+^ levels, we did not observe any different effect of TPC2-A1-N in all cell lines tested. TPC2-A1-N significantly and rapidly also increased intracellular Na^+^ in MEF WT and all our TPC KO lines. This result does not match with the postulated selectivity of TPC2-A1-N for Ca^2+^ release. In summary, we conclude that TPC2-A1-N exerts its Ca^2+^ increasing effects independent of any TPC. Effects were also independent of cell type and species.

TPC2-A1-N has been identified and verified in an artificial experimental setup and accordingly was postulated as a specific modulator of TPC2. The substance is also commercially available and is sold as a specific membrane-permeable TPC2 activator and as an NAADP mimetic. Therefore, it is expected that TPC2-A1-N penetrates the plasma membrane and activates the intracellular channel independent of experimental conditions or cell populations. However, our study shows that this general validity is not given. Possible reasons for these discrepancies may be limited accessibility of native TPC2 channel complexes for TPC2-A1-N caused by interactions with the NAADP-binding proteins JPT2 or LSM12. Since TPC2-A1-N presumably activates TPC2 independent of these NAADP-binding proteins and activates TPC2 in artificial membrane environments, this might be a possible explanation for the different observations.

Our studies indicate that TPC2-A1-N exerts its Ca^2+^ increasing effects in a target-specific way. In each of our experimental setups, application of ionomycin released residual Ca^2+^ from intracellular compartments that were unsusceptible for TPC2-A1-N. This observation suggests that TPC2-A1-N targets a specific, large intracellular Ca^2+^-store, presumably the ER and leaves distinct cellular compartments intact. This hypothesis is further supported by thapsigargin, a well-known substance that depletes Ca^2+^ from the ER. Application of thapsigargin causes a massive decrease in the Ca^2+^ mobilizing effect of TPC2-A1-N. Therefore, we assume that the ER has key role and represents the main source of Ca^2+^ released by TPC2-A1-N. In order to identify a potential role of IP_3_ receptors, we inhibited this receptor by addition of 2-APB and investigated the remaining Ca^2+^ mobilizing effects of histamine and TPC2-A1-N in this order and *vice versa*. Our results indicate that at least part of TPC2-A1-N–mediated Ca^2+^ release is independent of the IP_3_ receptor. The precise target of TPC2-A1-N is still unknown and has to be identified.

The usage of GCaMP constructs as additional and alternative Ca^2+^ measurement protocols substantiate our findings. This was shown for the release of Ca^2+^ from the ER with the ER-GCaMP6-210 construct and for visualizing cytosolic Ca^2+^ with the alternative GECI TPC1-GCaMP7f. We want to emphasize the fact that the results from the latter must not be misinterpreted in the way that the distribution of fluorescence is limited to the surrounding area of endo-lysosomes, but is determined by the targeted expression of this construct.

Our results do not reflect previous publications arguing for a role of TPC2-A1-N as an NAADP mimetic of TPC2. We strongly emphasize the fact that our data have been captured in a protocol that keeps TPCs in their natural endo-lysosomal membrane environment. Many sophisticated experimental approaches have been established in the literature for studying TPCs, but neglect the fact that they rely on numerous assumptions. In contrast to those approaches, our protocol does not disturb accessory subunit composition of TPCs, does not change intraorganellar pH or phospho-lipid composition, and does not create new artificial vacuolar membrane environments or uses mutant TPCs.

In summary, we claim that the small molecule activator TPC2-A1-N increases intracellular Ca^2+^ levels independent of any TPC and that the main Ca^2+^ source targeted by TPC2-A1-N is the ER.

## Experimental procedures

### Cell culture and generation of TPC KO cell lines

MEF and HeLa cells were cultivated in Dulbecco's modified Eagle's medium (DMEM) high glucose media (anprotec)—supplemented with 1 mM sodium pyruvate (PAA Laboratories), 1× MEM nonessential amino acids mix (anprotec), 1× Penicillin-Streptomycin solution (anprotec), and 10% fetal calf serum (FCS, anprotec)—at 5% CO_2_ and 37 °C. When cultures reached a confluence of 80 to 90%, cells were passaged using 0.05% Trypsin/0.02% EDTA in PBS without Ca^2+^ and Mg^2+^ (PAN-Biotech).

J774 cells were cultivated in DMEM high glucose media (anprotec), supplemented with 1× MEM nonessential amino acid mix (anprotec), 1× Penicillin-Streptomycin solution (anprotec), and 5% FCS (anprotec), at 5% CO_2_ and 37 °C. When cultures reached a confluence of 80 to 90%, cells were washed once in PBS without Ca^2+^ and Mg^2+^ (PAN-Biotech), dislodged from the flask by mechanical scraping and diluted 1 to 10 in new medium.

For imaging experiments, cells were seeded on poly-L-lysine–coated coverslips (Marienfeld Superior) 24 h before Ca^2+^ imaging experiments. CRISPR/Cas9-mediated generation of TPC1-, TPC2-, and TPC1/2-KO MEF cell lines was described in Castonguay *et al.*, 2017 and Müller *et al.*, 2021.

### CRISPR/Cas9 generated knockout of TPC1/2-KO in HeLa and J774 cells

The CRISPOR web tool (CRISPOR.org) was used to identify guide RNA-binding sites for Cas9-mediated genome editing (34). Inactivation of TPC genes in J774 cells was achieved by using the single guide RNAs CGTCCGGCACAAACGTACCA (TPC1 exon 5) and TGGCCTGACCGAGACGATCG (TPC2 exon 4). The single guide RNAs GATGTCCATGAAGGGCGGCA (TPC1 exon 8) and GACACTCTCGGTCAGGCCGC (TPC2 exon 4) were used to edit TPC genes in HeLa cells (Figs. S3, *A*, *B*, and S4, *A*, *B*).

Briefly, cells were transfected with pSpCas9(BB)-2A-Puro vectors containing either the sgRNA sequences targeting TPC1 or TPC2 and cultivated in puromycin containing selection media. After selection, cells were clonally expanded and checked for recombination events. TPC1/2-KO was confirmed by sequencing of genomic DNA and immunoblot analysis. Only cell clones were used further that showed the same edit on both alleles, that is, that resulted in an unambiguous sequence.

### Western blot analysis

Samples probed for TPCs were dissolved in 4 M urea containing SDS sample buffer (250 mM Tris/HCl, 12.5% glycerol, 2% SDS, 0.0005% bromophenol blue, 2.5% β-mercaptoethanol). The protein amount of samples was determined utilizing the RC DC Protein Assay Kit (Bio-Rad) and adjusted to an over-all protein concentration of 0.5 μg/μl. Proteins were separated by SDS-PAGE on 7% SDS-polyacrylamide gels. Separated proteins were transferred onto polyvinylidene fluoride membranes (Carl Roth) using a wet-electroblotting system (Trans-Blot© system, Bio-Rad). Membranes were incubated over night at 4 °C with the primary antibodies detecting TPC1 (6, 29), TPC2 (8) tubulin (Santa Cruz Biotechnology), or Na^+^/K^+^-ATPase (abcam). After incubation with horseradish peroxidase-coupled secondary antibodies, immunoblots were analyzed using a Pierce ECL Western blotting substrate kit (Thermo Fisher Scientific) and LAS-3000 mini-image system (Fujifilm).

For HeLa cells, we additionally performed a transfection with a plasmid encoding human TPC2 to prove suitability of TPC2 antibody for human cells.

### Fura-2 and CoroNa Green imaging experiments

Stock solution of Fura-2 AM (Abcam), CoroNa Green AM (Invitrogen) and Pluronic F127 (Invitrogen) were prepared following the manufacturers´ instructions. Ca^2+^ and Na^+^ imaging experiments were performed in HBSS-1 (Thermo Fisher Scientific) supplemented with 2 mM Ca^2+^ or HBSS-2 (HBSS without Ca^2+^). Fura-2 AM and CoroNa Green AM incubation, washing steps and imaging experiments were performed at room temperature. Cells were incubated in the dark for 1 h in 2 μM Fura-2 AM and 0,002% Pluronic F127 solved in DMEM high glucose medium without FCS. For CoroNa Green-AM experiments, cells were incubated for 40 to 45 min in the dark with the Na^+^ indicator solved in DMEM high glucose medium without FCS at a concentration of 5 μM. Coverslips carrying Fura-2 or CoroNa Green loaded cells were washed three times with 2 ml of HBSS-1 and mounted in a perfusion chamber. Cells were left for 10 to 15 min in the dark to allow de-esterification of the fluorophore-acetoxymethyl esters.

Immediately before start of Fura-2 Ca^2+^ measurements, cells were washed three times with HBSS-2 buffer. If not stated otherwise, all Fura-2 measurements were carried out in Ca^2+^-free buffer. If EGTA was added to remove any traces of Ca^2+^, cells were rinsed for 2 min with HBSS-2 at a flow rate of 15 ml/min to wash out chelated Ca^2+^. TPC2-A1-N (MedChemExpress), thapsigargin (Tocris bioscience), 2-APB (Tocris), and ionomycin (Thermo Fisher Scientific) were dissolved in DMSO (Invitrogen). Histamine (Sigma) was predissolved in HBSS. Aliquoted chemicals were stored at −20 °C according to the manufacturers´ instructions.

Image acquisition for Ca^2+^ and Na^+^ was performed with an Axiovert 200 inverted microscope (Zeiss) equipped with a 40×, 1.3 fluar oil objective and coupled to a pcoPanda 4.2 bi-sCMOS camera (PCO). Imaging experiments were performed with the LedHUB LED Light-Engine (Omicron Laserage) equipped with 340 nm, 385 nm, and 475 nm LEDs and corresponding excitation/bandpass filters (Chroma) of 340 nm, 380 nm, and 482 nm. Images were acquired every 3 s and emitted fluorescence intensities for Fura-2 (at 510 nm) or CoroNa Green (536 nm) were measured.

Single-cell Ca^2+^ and Na^+^ measurements were performed with VisiView (V5.0.0.4, Visitron Systems; https://www.visitron.de/products/visiviewr-software.html) imaging software. Baseline level was defined as the mean of 10 consecutive time points immediately before application of indicated substances.

Region of interests were assigned and analyzed for each cell of the image section. On average, 28 MEF cells, 30 J774 cells, and 30 HeLa cells were analyzed per experiment. To determine background fluorescence of cells, Fura-2 emission was quenched at the end of each experiment by adding manganese (25 mM). The background of each region of interest analyzed was subtracted from the determined Fura-2 signals.

### GCaMP calcium imaging

MEF-cells were seeded on poly-L-lysine–coated coverslips (Marienfeld Superior) 16 h before transfection with the ER calcium reporter ER-GCaMP6-210. Cells expressing the ER-GCaMP6 construct were analyzed 24 to 48 h after transfection. ER-GCaMP6-210 was a gift from Timothy Ryan (Addgene plasmid # 86919; http://n2t.net/addgene:86919; RRID:Addgene_86919) (33). Airyscan live-cell calcium imaging videos were generated using a LSM800 (Zeiss) confocal laser-scanning microscope equipped with a 63×, 1.4 NA oil objective, Airyscan detector 2.0 (Zeiss), PeCon incubation chamber (37 °C, 5% CO2) and Zen blue software (Zeiss; https://www.zeiss.com/microscopy/de/produkte/software/zeiss-zen.html). Time-lapse videos were further processed by Zen blue and Imaris software (https://imaris.oxinst.com/). Transfected cells were washed three times with HBSS substituted with 2 mM CaCl_2_ and NucBlue was added to the cells 20 min prior imaging. Time interval between images/frames was set to 30 s. The imaging protocol was carried out as followed: frames 0 to 5 (0–2 min) were imaged untreated to account for potential bleaching, before 0.2% DMSO was added to the cells after frame 5. DMSO control treatment lasted from frames 6 to 10 (2:30–4:30 min) before 20 μM TPC2-A1-N was added after frame 10. TPC2-A1-N treatment lasted from frames 11 to 20 (5:00–9:30 min) until ER-GCaMP fluorescence was almost undetectable. A total of 2.5 μM ionomycin was applied to the cells after frame 20 until ER-GCaMP fluorescence was visually reduced (Frame 21-endpoint video; 10:00 min-end).

For endo-lysosomal Ca^2+^ imaging of TPC1-GCaMP7f transfected MEF cells, cells were washed three times with Ca^2+^-free HBSS and NucBlue was added to the cells 20 min prior imaging. Time interval between images/frames was set to 30 s. For TPC1-GCaMP7f imaging the protocol was carried out as followed: frames 0 to 5 (0–2 min) were imaged untreated to account for potential bleaching before 20 μM TPC2-A1-N was applied to the cells lasting from frame 5 to 10 (2:30–4:30 min). A total of 2.5 μM ionomycin was applied to the cells after frame 10 until frame 15 (5:00–7:00 min), before Ca^2+^ was added to achieve a final concentration of 2 mM (7:30 min-end).

### Statistical and data analysis

The statistical analyses of this study were conducted using GraphPad Prism version 10.5.0 for Windows (GraphPad Software, www.graphpad.com). Two-way repeated measures ANOVA were applied if dependent variables over two or more time points were analyzed. One-way ANOVA was used to test for differences among three or more groups of datasets, Student’s *t* test if only two means were compared. *p* values < 0.05 were considered to be significant.

## Data availability

All primary data are available from the corresponding author upon reasonable request. No data have been deposited into publicly accessible repositories.

## Supporting information

This article contains the following supporting information: Fig. S1–S11 and videos V1–V6.

## Conflict of interest

The authors declare that they have no conflicts of interest with the contents of this article.
