# Supplementary material for: Small molecule agonist TPC2-A1-N increases intracellular Ca2+ independent of two-pore channels
Source: J Biol Chem. 2025 Aug 8;301(9):110576. doi: 10.1016/j.jbc.2025.110576 (PMC12446622; doi:10.1016/j.jbc.2025.110576)
Supplement: Supporting Figures [file mmc1.docx]

**Small molecule agonist TPC2-A1-N increases intracellular Ca^2+^ independent of Two-Pore Channels**

Robert T. Mallmann^1^, Marlene C. Gonzalez Mantuano^1^, Katharina Polomski^1^, Julian Knerr^1^ and Norbert Klugbauer^1^

^1^Institut für Experimentelle und Klinische Pharmakologie und Toxikologie, Medizinische Fakultät, Albert-Ludwigs-Universität Freiburg, Germany;

**Supporting information:**

SI figures S1 to S11

SI videos V1 to V6

**SI figures and legends**

**
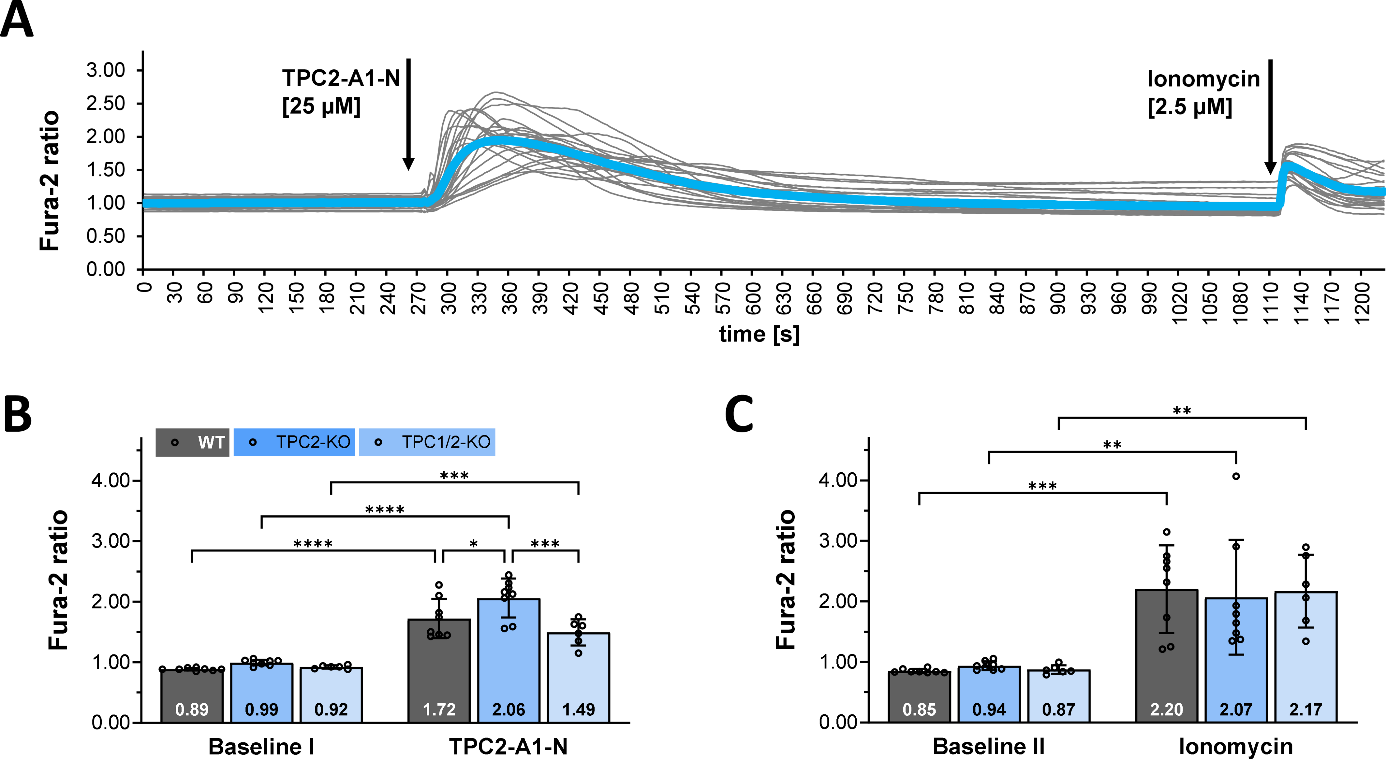
**

**Fig. S1: Ca^2+^ release from intracellular stores in MEF cells following addition of 25 µM TPC2-A1-N.** (A) Single track recordings from 23 Fura-2 loaded TPC2-KO cells (grey lines correspond to each individual cell, MEAN Fura-2 ratio is shown as blue line). Fura-2 ratios were gathered in Ca^2+^ free HBSS buffer. Following incubation with TPC2-A1-N 2.5 µM ionomycin was added. (B) Comparison of Fura-2 ratios at baseline levels and maximal Fura-2 ratios following treatment with TPC2-A1-N. (C) Comparison of Fura-2 ratios at baseline levels and maximal Fura-2 ratios after application of ionomycin.

Mean values represent the average maximal Fura-2 ratios of N experiments per cell line (MEF-WT (N=8), MEF-TPC2-KO (N=8) MEF-TPC1/2-KO (N=6); N=number of experiments). Data are presented as MEAN ± SD. Two-Way Repeated Measures ANOVA followed by Bonferroni`s multiple comparisons test; * p<0.05, ** p<0.01, *** p<0.001, **** p<0.0001.

**
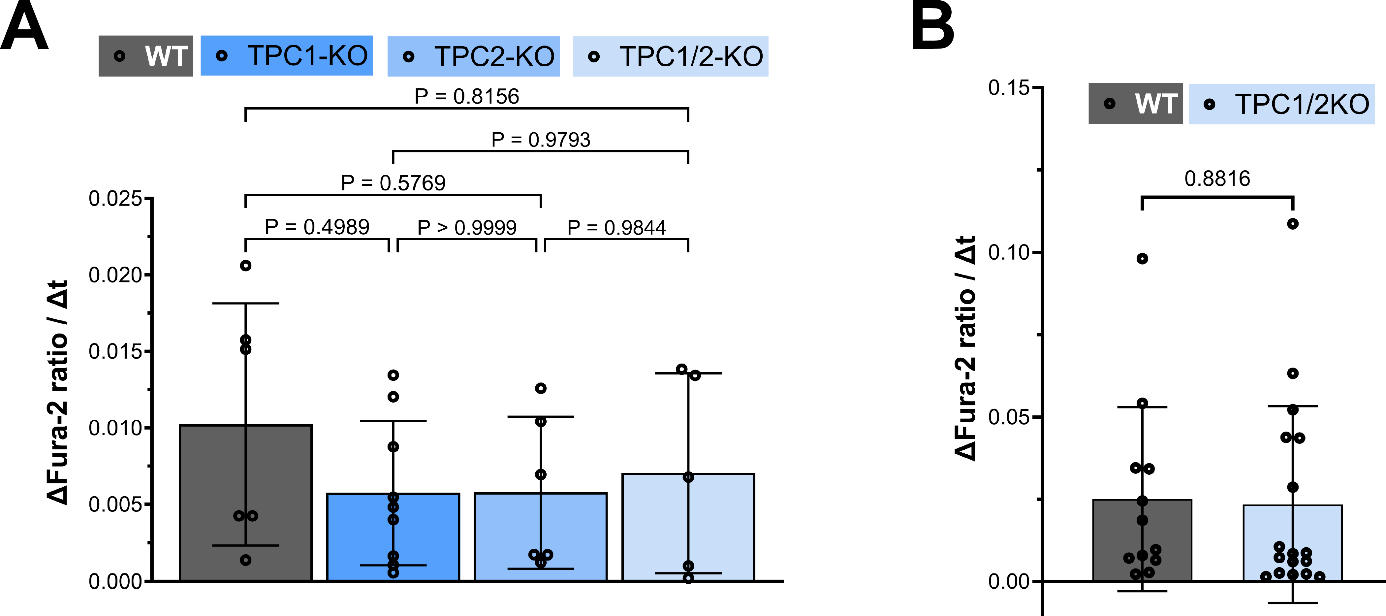
**

**Fig. S2: Slopes of the Fura-2 ratios over time at the half-maximum values following application of TPC2-A1-N.** (A) Fura-2 ratios over time at the half-maximum values for WT and TPC-deficient MEF cells following application of 10 µM TPC2-A1-N. Dataset is from main figure 1. Mean values represent the average slopes of Fura-2 ratios over time at the half-maximum. Data are presented as MEAN ± SD. N experiments per cell line: MEF-WT (N=6), MEF-TPC1-KO (N=9), MEF-TPC2-KO (N=6) and MEF-TPC1/2-KO (N=5); One-Way ANOVA and Tukey’s multiple comparisons test. (B) Fura-2 ratios over time at the half-maximum values for WT and TPC1/2-deficient MEF cells in the presence of 0.5 mM EGTA and following application of 10 µM TPC2-A1-N. Dataset is from main figure 2. Data are presented as MEAN ± SD. Mean values represent the average slopes of Fura-2 ratios over time at the half-maximum. N experiments per cell line: MEF-WT (N=12) and MEF-TPC1/2KO (N=18); Unpaired Student’s t-test.


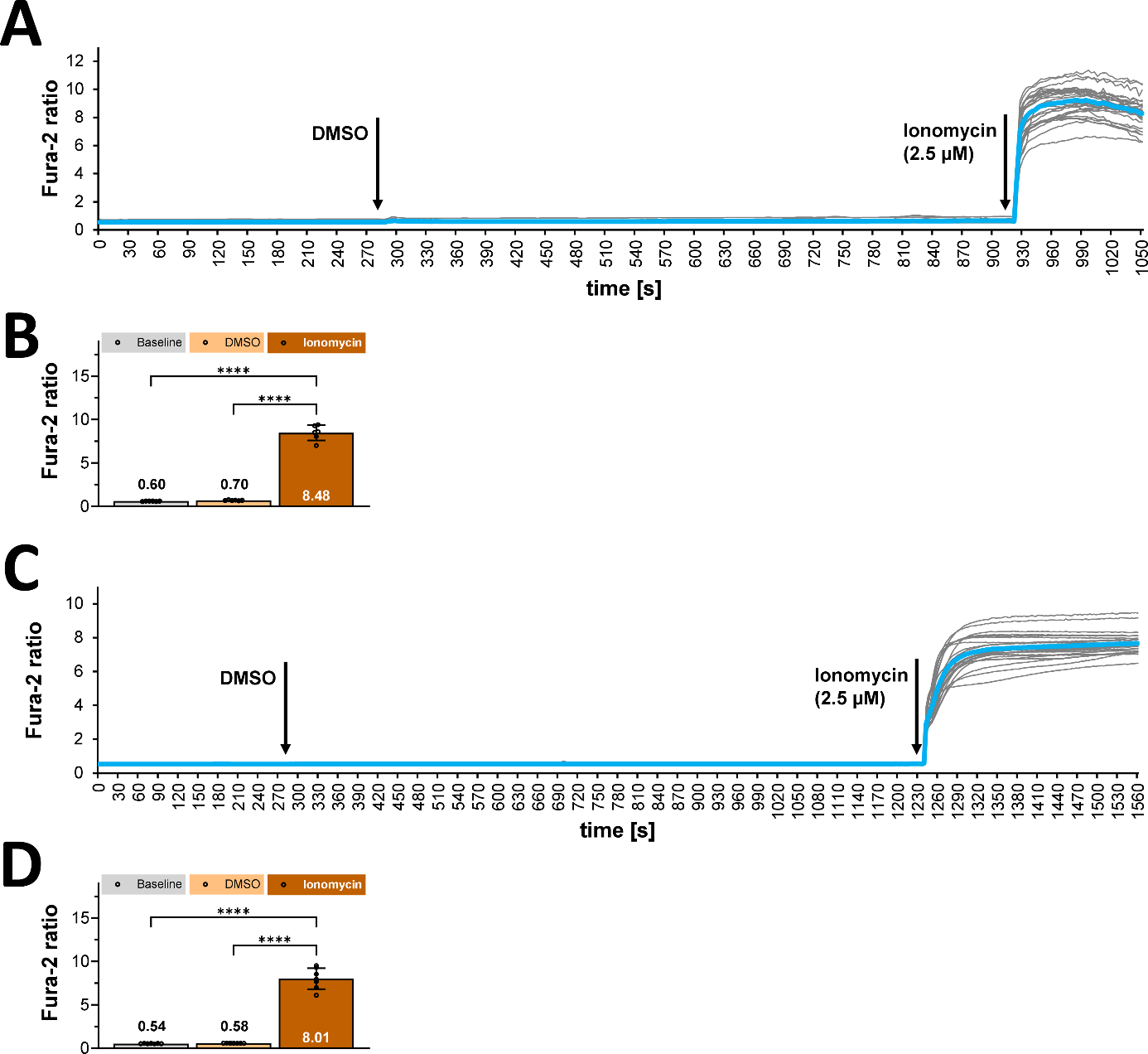


**Fig. S3: DMSO control for Ca^2+^ release experiments in MEF and HeLa wildtype cells.** (A) Single track recordings from 30 Fura-2 loaded MEF-WT cells (grey lines correspond to each individual cell, MEAN Fura-2 ratio is shown as blue line). DMSO (0.25 %) was used in the same concentration as for 25 µM TPC2-A1-N experiments. Fura-2 ratios were gathered in HBSS buffer supplemented with 2 mM Ca^2+^. Following incubation with DMSO, 2.5 µM ionomycin was added. (B) Comparison of Fura-2 ratios at baseline levels before and maximal changes of Fura-2 ratios after application of DMSO and ionomycin. (C) Single track recordings from 23 Fura-2 loaded HeLa-WT cells (grey lines correspond to each individual cell, MEAN Fura-2 ratio is shown as blue line). DMSO (0.6 %) was used in the same concentration as for 60 µM TPC2-A1-N experiments. Fura-2 ratios were gathered in HBSS buffer supplemented with 2 mM Ca^2+^. Following incubation with DMSO 2.5 µM ionomycin was added. (D) Comparison of Fura-2 ratios at baseline levels before and maximal changes of Fura-2 ratios after application of DMSO and ionomycin. Mean values represent the average Fura-2 ratios of 6 (MEF) and 7 (HeLa) experiments. Data are presented as MEAN ± SD. One-Way ANOVA followed by Tukey’s multiple comparisons test; **** p<0.0001.


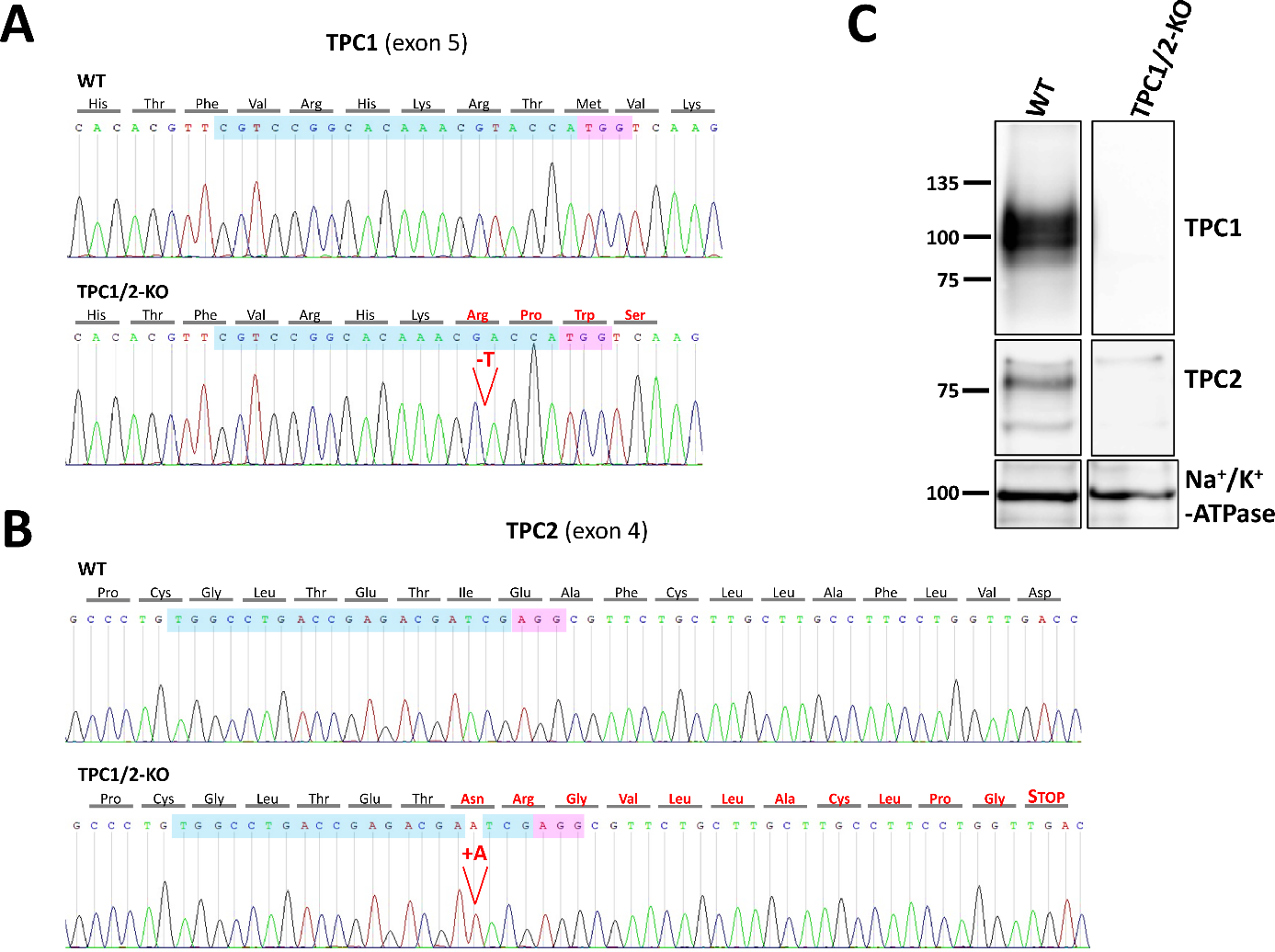


**Fig. S4: CRISPR/Cas9 mediated genetic inactivation of TPC1 and TPC2 in J774 cells.** (A) and (B) Strategy for generation and sequence validation of a TPC1/2 double knockout J774 cell line. Localization of gRNA binding sites (pale blue) and corresponding PAM sequences (pale pink) located in exon 5 for TPC1 (A) and in exon 4 for TPC2 (B), respectively. Cas9 induced recombination resulted in a one base pair deletion in exon 5 of both alleles of the TPC1 gene and a one base pair addition in exon 4 of both alleles of the TPC2 gene. Both mutations cause frame shifts and premature stop codons. (C) Inactivation of TPC1 and TPC2 genes was confirmed by Western blots.


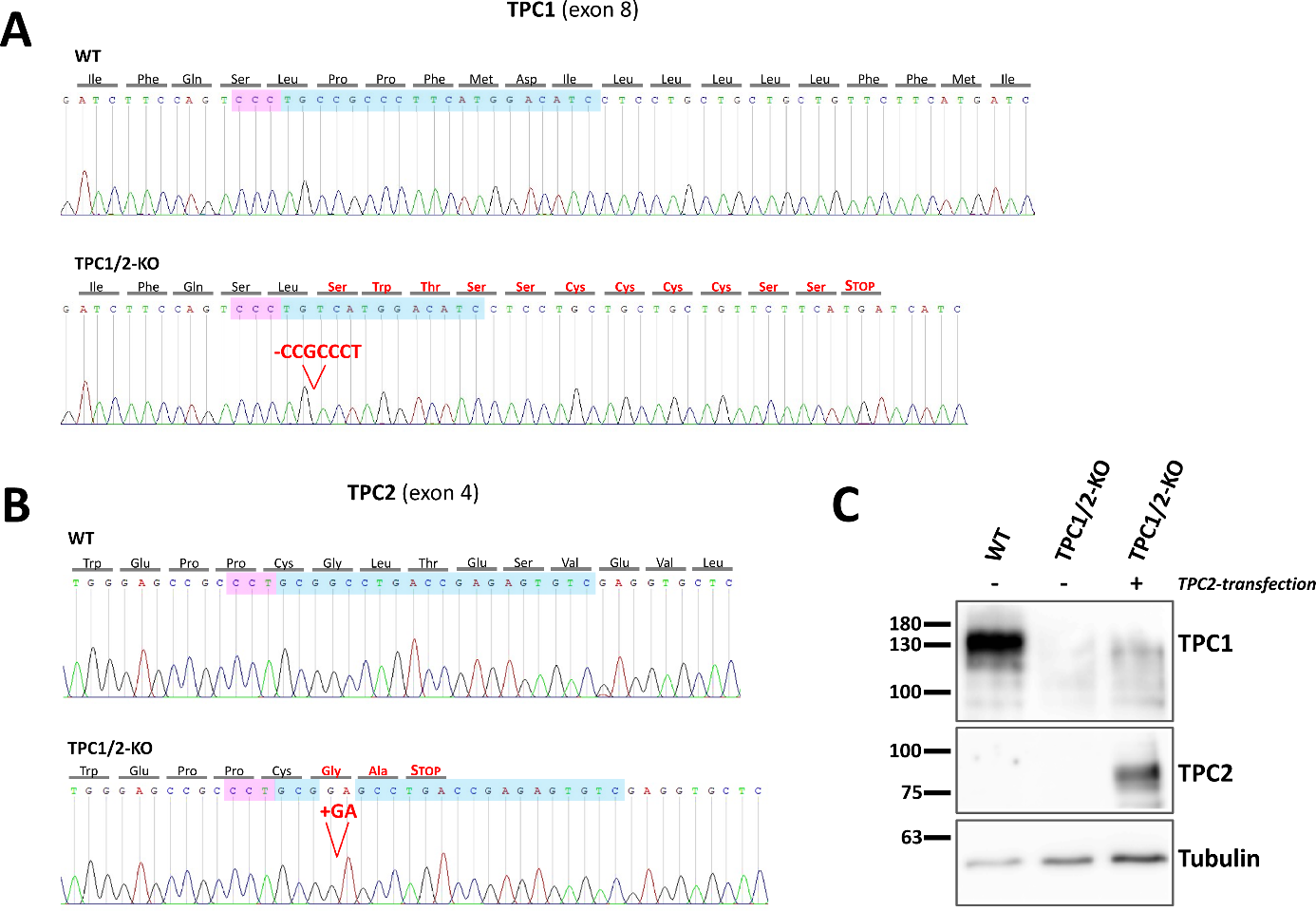


**Fig. S5: CRISPR/Cas mediated genetic inactivation of TPC1 and TPC2 in HeLa cells.** (A) Strategy for generation and sequence validation of TPC1 knock-out in exon 8. Localization of gRNA binding sites (pale blue) and corresponding PAM sequences (pale pink). Cas9 induced recombination resulted in deletion of 7 base pairs in both alleles of the TPC1 gene, thereby causing a frame shift and a premature stop codon. (B) Strategy for generation and sequence validation of TPC2 knock-out in exon 4. Cas9 induced recombination resulted in addition of 2 base pairs in both alleles of the TPC2 gene, thereby causing a frame shift and a premature stop codon. (C) Inactivation of TPC1 and TPC2 genes was confirmed by Western blot. Please note that expression of TPC2 in wildtype HeLa cells is already below detection limit (left lane). To confirm detection of human TPC2 by the antibody, we transfected HeLa TPC1/2 double knock-out cells with a plasmid encoding human TPC2. Western blot analysis of transfected cells resulted in a protein band of correct size (right lane).


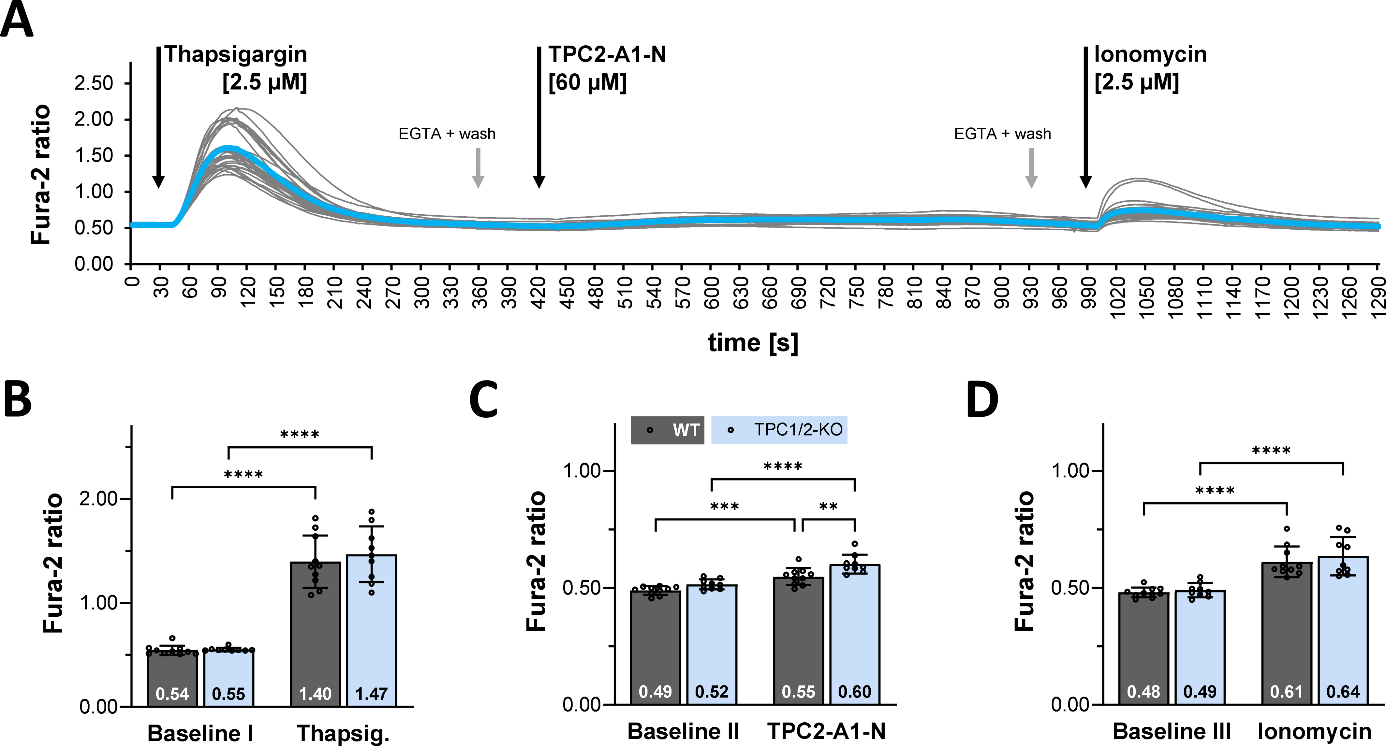


**Fig. S6: Reduced TPC2-A1-N evoked Ca^2+^ signal in HeLa cells after ER Ca^2+^ depletion by thapsigargin.** (A) Single track recordings from 30 Fura-2 loaded HeLa-TPC1/2-KO cells (grey lines correspond to each individual cell, MEAN Fura-2 ratio is shown as blue line). Cells were incubated first in HBSS supplemented with 2 mM CaCl_2_. Fura-2 ratios were analyzed following buffer-exchange to Ca^2+^ free HBSS. EGTA (0.5 mM) and a washing step with Ca^2+^ free HBSS buffer were applied between each stimulation step to remove all residual extracellular Ca^2+^. Following incubation with 60 µM TPC2-A1-N 2.5 µM ionomycin was added to asses for Ca^2+^ release from any intracellular store that was not targeted either by thapsigargin or TPC2-A1-N. (B) Comparison of Fura-2 ratios at baseline levels and maximal Fura-2 ratios following treatment with 2.5µM thapsigargin. (C) Comparison of Fura-2 ratios at baseline levels and maximal Fura-2 ratios following treatment with TPC2-A1-N. (D) Comparison of Fura-2 ratios at baseline levels and maximal Fura-2 ratios after application of ionomycin. HeLa-WT (N=10) HeLa-TPC1/2KO (N=9); N=number of experiments. Data are presented as MEAN ± SD. Two-Way Repeated Measures ANOVA followed by Bonferroni’s multiple comparisons test; ** p<0.01, *** p<0.001, **** p<0.0001.

**
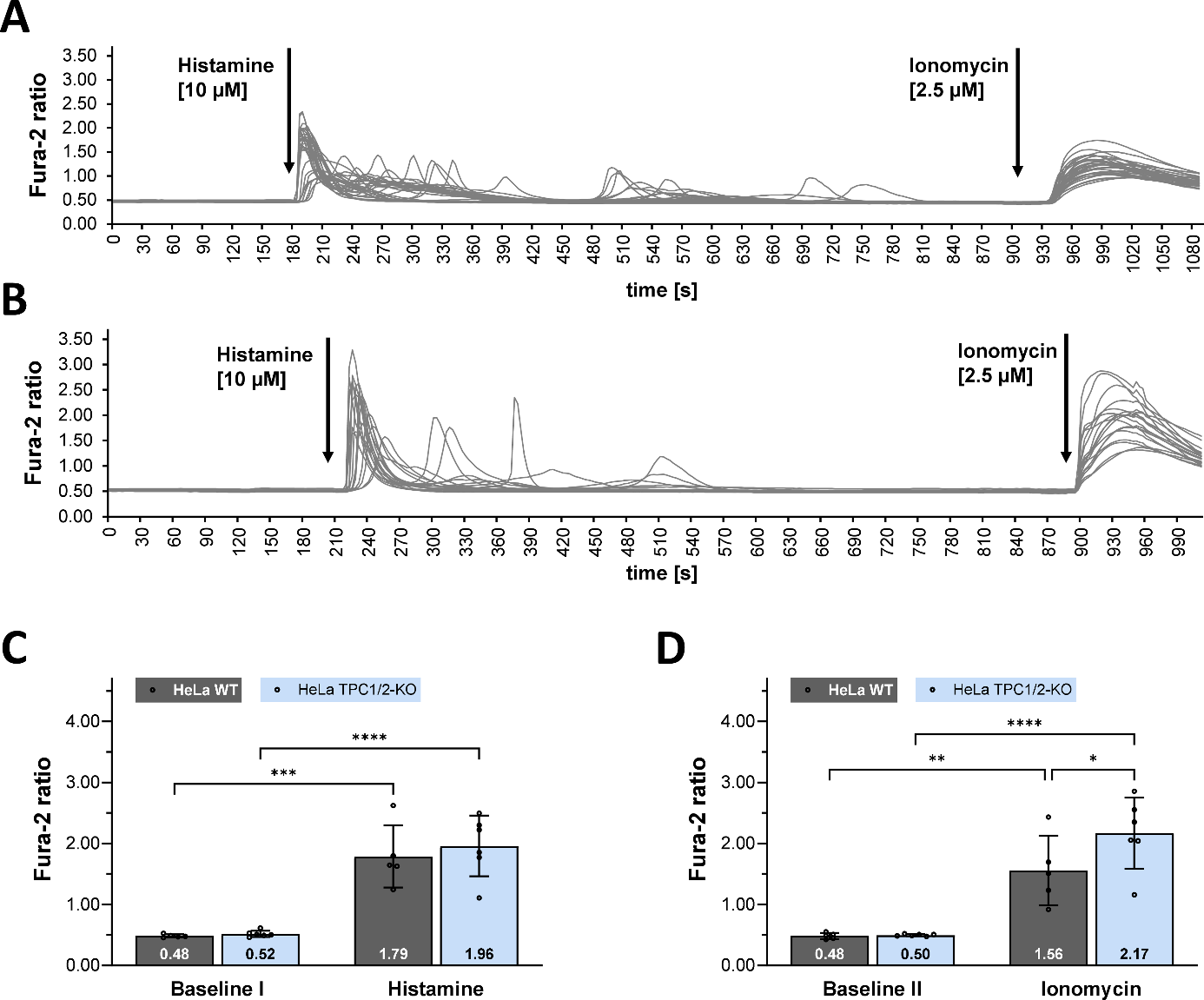
**

**Fig. S7: Histamine evoked Ca^2+^ signals in HeLa wildtype and TPC1/2 knock-out cells.** (A) Single track recordings from 30 Fura-2 loaded wildtype HeLa cells (grey lines correspond to each individual cell). Fura-2 ratios were gathered in Ca^2+^ free HBSS buffer. Following incubation with histamine, ionomycin was added as indicated. (B) Single track recordings from 18 Fura-2 loaded TPC1/2-KO HeLa cells (grey lines correspond to each individual cell). Fura-2 ratios were gathered in Ca^2+^ free HBSS buffer. Following incubation with histamine, ionomycin was added as indicated. (C) Comparison of Fura-2 ratios at baseline levels and maximal Fura-2 ratios following treatment with histamine. (D) Comparison of Fura-2 ratios at baseline levels and maximal Fura-2 ratios after application of ionomycin.

Mean values represent the average maximal Fura-2 ratios of N experiments per cell line: (HeLa-WT (N=5), HeLa-TPC1/2-KO (N=6); data are presented as MEAN ± SD. Two-Way Repeated Measures ANOVA followed by Bonferroni`s multiple comparisons test; * p<0.05, ** p<0.01, *** p<0.001, **** p<0.0001.

**
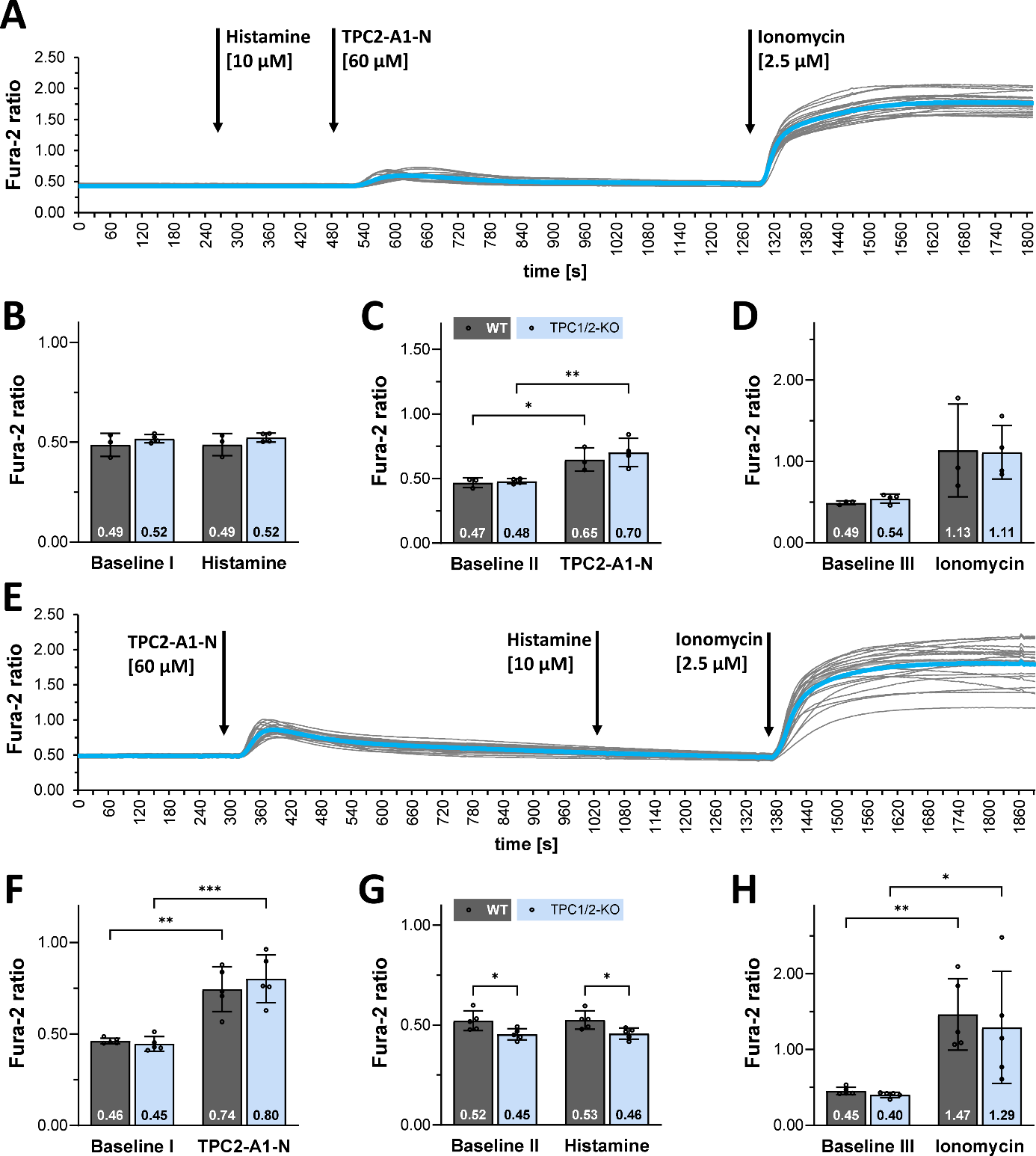
**

**Fig. S8: Inhibition of IP_3_ receptors by 2-APB abolishes histamine, but not TPC2-A1-N evoked Ca^2+^ signals in HeLa cells.** (A) Single track recordings from 21 Fura-2 loaded wildtype cells (grey lines correspond to each individual cell, MEAN Fura-2 ratio is shown as blue line). Fura-2 ratios were gathered in Ca^2+^ free HBSS buffer. Following incubation with 100 µM 2-APB histamine, TPC2-A1-N and ionomycin were added as indicated. (B) Comparison of Fura-2 ratios at baseline levels and maximal Fura-2 ratios following treatment with histamine. (C) Comparison of Fura-2 ratios at baseline levels and maximal Fura-2 ratios after application of TPC2-A1-N. (D) Comparison of Fura-2 ratios at baseline levels and maximal Fura-2 ratios after application of ionomycin. Mean values represent the average maximal Fura-2 ratios of N experiments per cell line (WT (N=3), TPC1/2-KO (N=4)) (E) Single track recordings from 21 Fura-2 loaded wildtype cells (grey lines correspond to each individual cell, MEAN Fura-2 ratio is shown as blue line). Following incubation with 100 µM 2-APB TPC2-A1-N, histamine and ionomycin were added as indicated. (F) Comparison of Fura-2 ratios at baseline levels and maximal Fura-2 ratios following treatment with TPC2-A1-N. (G) Comparison of Fura-2 ratios at baseline levels and maximal Fura-2 ratios after application of histamine. (H) Comparison of Fura-2 ratios at baseline levels and maximal Fura-2 ratios after application of ionomycin. Mean values represent the average maximal Fura-2 ratios of N experiments per cell line (WT (N=5), TPC1/2-KO (N=5)). Data are presented as MEAN ± SD. Two-Way Repeated Measures ANOVA followed by uncorrected Fisher`s LSD test; * p<0.05, ** p<0.01, *** p<0.001.

**
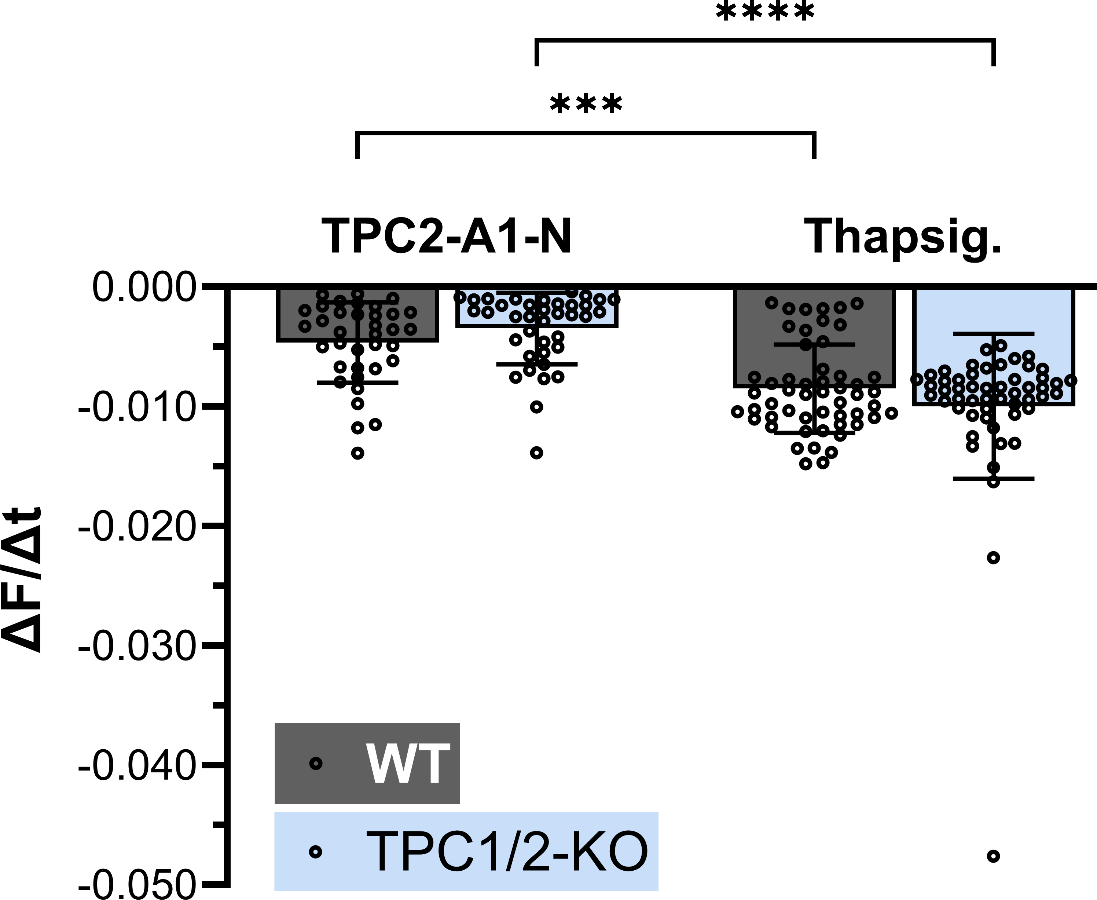
**

**Fig. S9:** Slopes at the half-minimal ΔF/Δt values after treatment with either 25 µM TPC2-A1-N or 2.5 µM thapsigargin. WT and TPC1/2-KO MEF cells were transiently transfected with ER-GCaMP6-210. Datasets correspond to main figure 5. Data are presented as MEAN ± SD. Two-Way ANOVA followed by Šidák multiple comparisons test; *** p<0.001, **** p<0.0001.

**
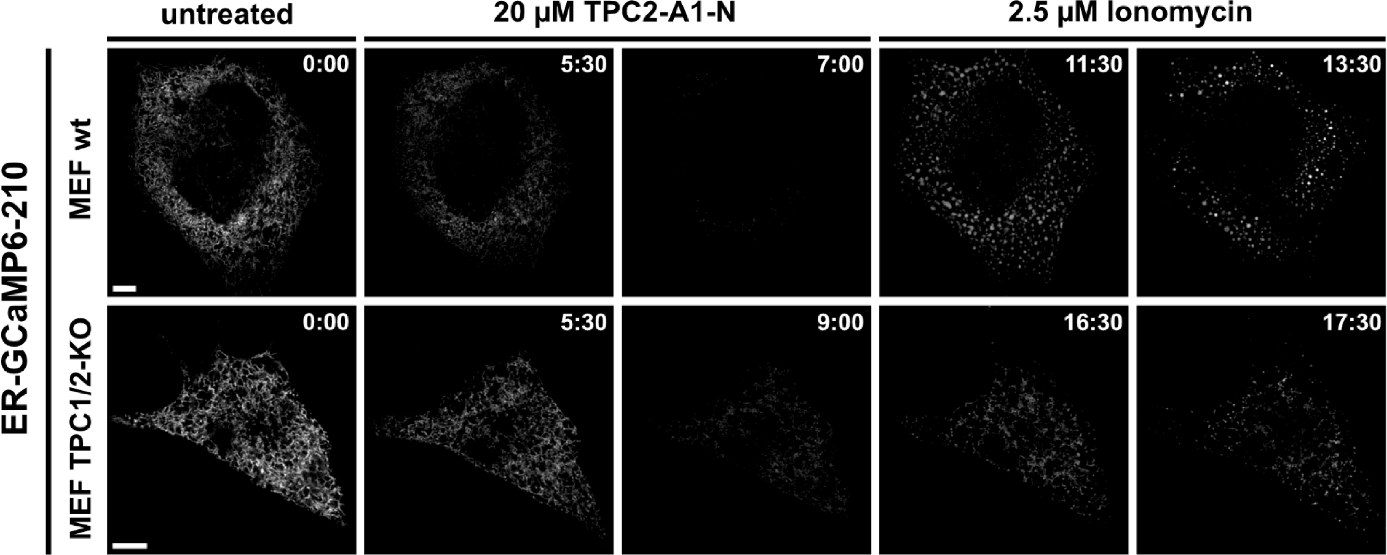
**

**Fig. S10: Additional example images from airyscan time-lapse videos of WT and TPC1/2-KO MEF cells transiently transfected with an ER targeted GCaMP-construct (ER-GCaMP-210, white).** Image series shows initially untreated cells (0-2 min), addition of 20 µM TPC2-A1-N until ER-GCaMP-210-fluorescence was almost undetectable (5:00-9:30 min) and addition of 2.5 µM ionomycin (10:00 min-end). Scale bar: 5 µm; time: mm:ss.

**A**


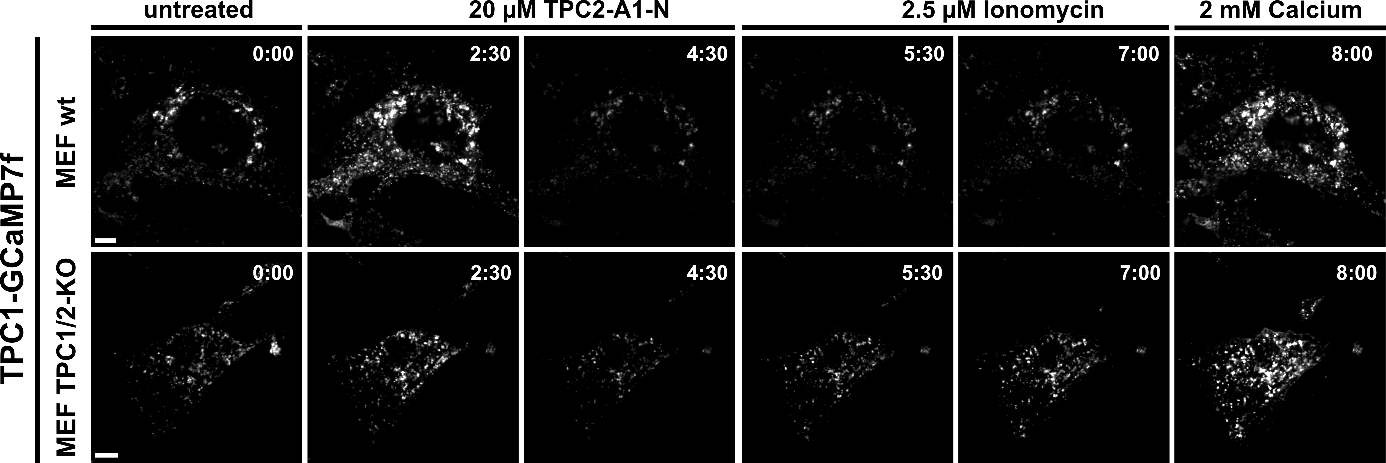


**B**

**
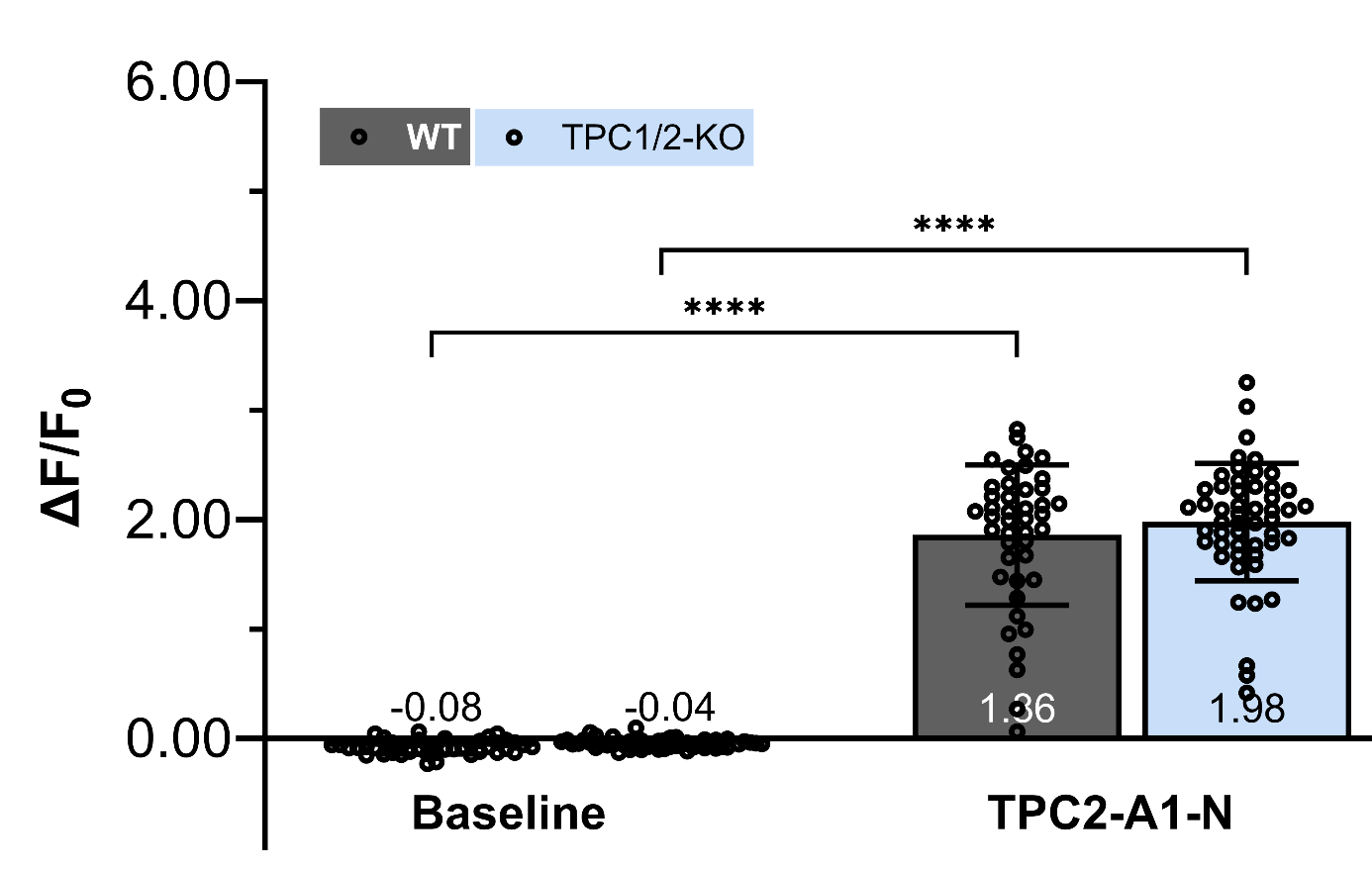
**

**Fig. S11: Example images from airyscan time-lapse videos of WT and TPC1/2-KO MEF cells transiently transfected with** **TPC1-GCaMP7f (white) and treated with TPC2-A1-N, ionomycin and Ca^2+^.** (A) Image series shows initially untreated cells (0-2 min), addition of 20 µM TPC2-A1-N (2:30-4:30 min), addition of 2.5 µM ionomycin (5:00-7:00 min), before Ca^2+^ was added to achieve a final concentration of 2 mM (7:30 min - end). Scale bar: 5 µm; time: mm:ss. (B) Comparison of WT and TPC1/2-KO MEF-cells transiently transfected with TPC1-GCaMP7f. Changes in fluorescence at baseline and maximal ΔF/F0 values following treatment with 20 µM TPC2-A1-N (MEF WT N=43 cells, 8 independent experiments; MEF TPC1/2-KO N=51 cells, 7 independent experiments). TPC1-GCaMP7f fluorescence was analyzed in Ca^2+^-free HBSS buffer. Data are presented as MEAN ± SD. Two-Way Repeated Measures ANOVA followed by Bonferroni`s multiple comparisons test; **** p<0.0001.

**Supporting information videos V1 to V6**

Video V1: Time-lapse videos of WT MEF cell transiently transfected with ER-GCaMP-210 (white) and treated with TPC2-A1-N and ionomycin (example images in fig. 5E).

Video V2: Time-lapse videos of TPC1/2-KO MEF cell transiently transfected with ER-GCaMP-210 (white) and treated with TPC2-A1-N and ionomycin (example images in fig. 5E).

Video V3: Time-lapse videos of WT MEF cell transiently transfected with ER-GCaMP-210 (white) and treated with TPC2-A1-N and ionomycin (example images in fig. S10).

Video V4: Time-lapse videos of TPC1/2-KO MEF cell transiently transfected with ER-GCaMP-210 (white) and treated with TPC2-A1-N and ionomycin (example images in fig. S10).

Video V5: Time-lapse videos of WT MEF cell transiently transfected with TPC1-GCaMP7f (white) and treated with TPC2-A1-N, ionomycin and Ca^2+^ (example images in fig. S11).

Video V6: Time-lapse videos of TPC1/2-KO MEF cell transiently transfected with TPC1-GCaMP7f (white) and treated with TPC2-A1-N, ionomycin and Ca^2+^ (example images in fig. S11).
